# Localization of Epileptic Foci Based on Simultaneous EEG–fMRI Data

**DOI:** 10.3389/fneur.2021.645594

**Published:** 2021-04-27

**Authors:** Seyyed Mostafa Sadjadi, Elias Ebrahimzadeh, Mohammad Shams, Masoud Seraji, Hamid Soltanian-Zadeh

**Affiliations:** ^1^Control and Intelligent Processing Center of Excellence (CIPCE), School of Electrical and Computer Engineering, College of Engineering, University of Tehran, Tehran, Iran; ^2^Neuroimage Signal and Image Analysis Group, School of Cognitive Sciences, Institute for Research in Fundamental Sciences (IPM), Tehran, Iran; ^3^Neural Engineering Laboratory, Department of Electrical and Computer Engineering, George Mason University, Fairfax, VA, United States; ^4^Center for Molecular and Behavioral Neuroscience, Rutgers University, Newark, NJ, United States; ^5^Behavioral and Neural Sciences Graduate Program, Rutgers University, Newark, NJ, United States; ^6^Medical Image Analysis Laboratory, Departments of Radiology and Research Administration, Henry Ford Health System, Detroit, MI, United States

**Keywords:** EEG-fMRI, epilepsy, localization, seizure onset zone, epileptic foci, BOLD response, IED

## Abstract

Combining functional magnetic resonance imaging (fMRI) and electroencephalography (EEG) enables a non-invasive investigation of the human brain function and evaluation of the correlation of these two important modalities of brain activity. This paper explores recent reports on using advanced simultaneous EEG–fMRI methods proposed to map the regions and networks involved in focal epileptic seizure generation. One of the applications of EEG and fMRI combination as a valuable clinical approach is the pre-surgical evaluation of patients with epilepsy to map and localize the precise brain regions associated with epileptiform activity. In the process of conventional analysis using EEG–fMRI data, the interictal epileptiform discharges (IEDs) are visually extracted from the EEG data to be convolved as binary events with a predefined hemodynamic response function (HRF) to provide a model of epileptiform BOLD activity and use as a regressor for general linear model (GLM) analysis of the fMRI data. This review examines the methodologies involved in performing such studies, including techniques used for the recording of EEG inside the scanner, artifact removal, and statistical analysis of the fMRI signal. It then discusses the results reported for patients with primary generalized epilepsy and patients with different types of focal epileptic disorders. An important matter that these results have brought to light is that the brain regions affected by interictal epileptic discharges might not be limited to the ones where they have been generated. The developed methods can help reveal the regions involved in or affected by a seizure onset zone (SOZ). As confirmed by the reviewed literature, EEG–fMRI provides information that comes particularly useful when evaluating patients with refractory epilepsy for surgery.

## Introduction

Localization of the epileptic generators is one of the striking topics in the treatment of epilepsy. It is still a challenge to find the precise brain regions of epileptic foci. Simultaneous EEG and fMRI data recordings are two modalities that can expose the brain regions with changes in metabolism and blood flow in response to epileptic spikes seen in the EEG, which are presumably accordant to the origin of epileptic discharges. fMRI which has a relatively poor temporal resolution but excellent spatial resolution is proper for localizing the brain regions with neuronal activity changes compared to the sham. This change is accompanied by a modification of the ratio of the concentration of oxy- and deoxy-hemoglobin in the blood, measured through the blood oxygen level-dependent (BOLD) effect (1, 2). In contrast, EEG has a high temporal resolution that makes it capable of measuring the neuronal currents directly from the scalp in the range of milliseconds but poor spatial resolution, which causes difficulty in determining the exact location of the current sources. The limitations of EEG are the deficiency in precise information of individual geometry and conductivity and the limited number of recording channels. Therefore, simultaneous recording of EEG and fMRI data provides a useful tool in using the two techniques' complementary features and overcoming the spatial limitations of EEG and fMRI's temporal boundaries.

An area where EEG and fMRI modalities have considerable clinical relevance is the pre-surgical evaluation in patients with epilepsy. In many patients with drug-resistant focal epilepsy undergoing surgery, standard magnetic resonance imaging (MRI) scans cannot visualize an exact source of epileptic seizures. Therefore, an invasive stereo-EEG analysis is required. However, simultaneous EEG and fMRI recordings offer a non-invasive alternative that can be a valuable approach for the localization of brain regions generating interictal epileptiform activity. This recording approach has become a useful tool for exploring ictal and interictal epileptic activity to reveal the epileptic foci and specify the relationship between hemodynamic changes and epileptic activity (3–6). EEG and fMRI are complementary for the localization of epileptic spike areas, but they can indicate different activity regions. Also, SEEG measures confirm EEG and fMRI results, although the concordance of simultaneous EEG–fMRI is not as good as the concordance between either one and SEEG (7). Unlike the general fMRI studies involving sensory, motor, and cognitive functions, the control and experimental conditions are determined based on the task. In epilepsy studies, these conditions are determined based on the absence and presence of epileptic discharges on the baseline of the EEG signal. So, in this context, the EEG signal is necessary for the analysis of fMRI data. The epileptic analysis of EEG–fMRI data is conventionally based on the identification of IEDs on EEG to create a regressor representing the effects of interest for a GLM analysis. Also, the model of epileptic activity is generally obtained by the convolution of EEG events as the stick functions of unitary amplitude with a predefined model of the event-related fMRI response, represented by the HRF. Finally, the activity maps showing the regions of significant IED-related change are obtained through the voxel-wise fitting of the model and application of appropriate statistical thresholds (3, 6, 8–10). Generally, BOLD responses are much less visible in patients with focal epilepsy compared to patients with generalized epilepsy (11, 12). Also, the posterior head regions are almost as involved as frontal regions in the BOLD response of patients with generalized epilepsy (11).

**Figure 1:**
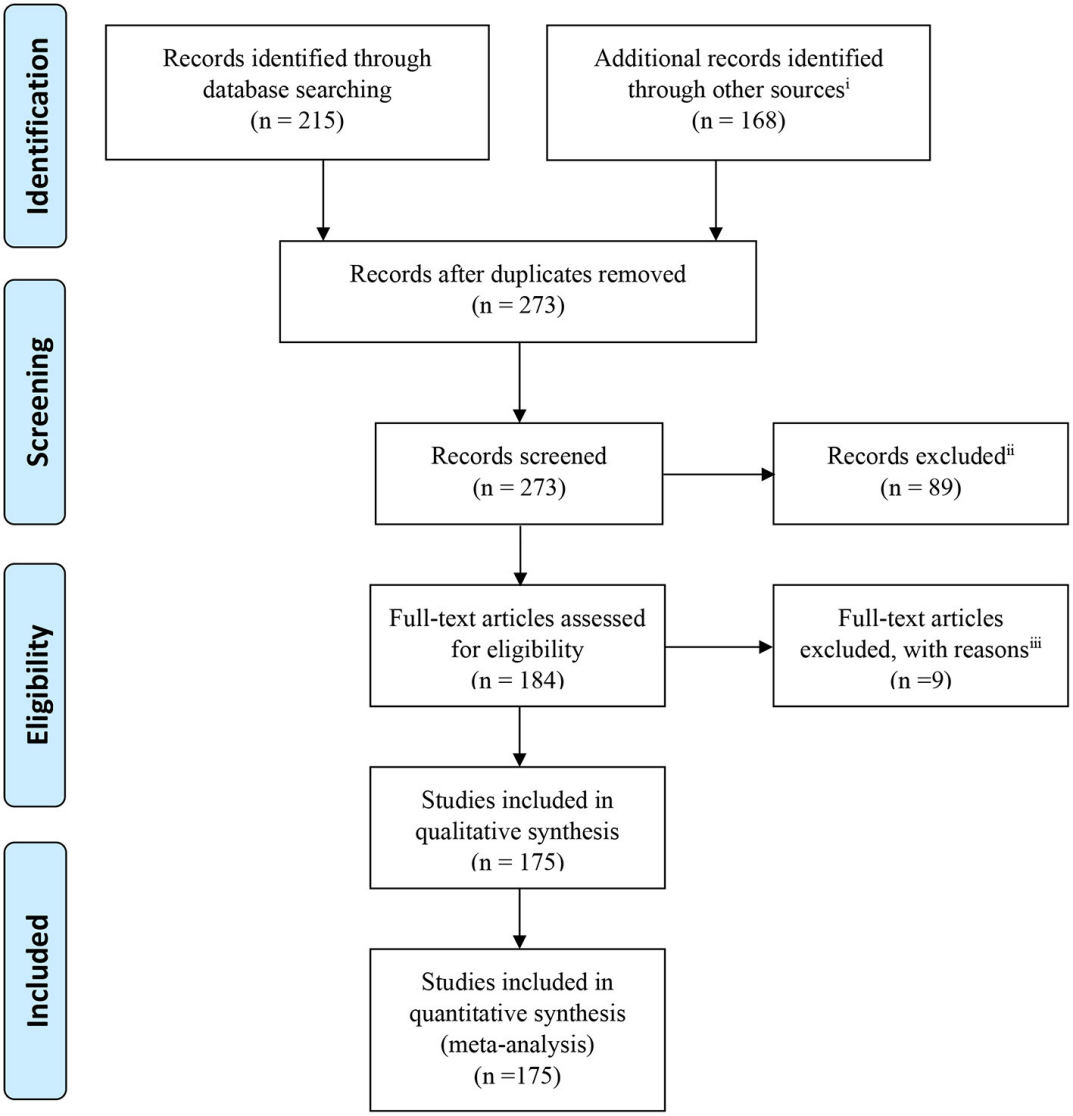
PRISMA flowchart showing the classification of the extracted articles related to the epileptic focus localization through simultaneous EEG–fMRI recording. ^i^Web of Science, Google Scholar, Pub Med, Scopus dataset, and ResearchGate websites and the references of the research and review articles. ^ii^Not relevant to research question, aims and objectives, or old articles. ^iii^Intracranial or ictal studies.

This paper reviews majority of the interictal studies presented with the aim of epileptic focus localization. For this purpose, the articles were classified based on their analysis method and reviewed in each part sorted by their publication date to reveal the trend of works in all the covered methods. First, we will present the primary concepts of epileptic source localization and analyze EEG inside the MRI scanner covered by associated studies. We will then review the localization methods and their clinical results obtained from patients with various types of epilepsy, showing the capability of each method for the pre-surgical evaluation of patients with epilepsy in comparison with the other methods. Finally, we discuss the complex issue of interpreting the result of EEG–fMRI in epilepsy studies. In this review, we tried to strike a balance between method-based studies and clinical outcomes. The Preferred Reporting Items for Systematic Reviews and Meta-Analyses (PRISMA) flowchart below shows the organization of the extracted articles (Figure 1). This review includes all EEG–fMRI studies focused on epileptic focus localization, which we have found by searching the related keywords in Google Scholar, PubMed dataset, Scopus, and ResearchGate. All studies are done interictally.

## Primary Concepts

### Signal Quality and Pre-processing

Recording EEG in the MR scanner requires non-magnetic electrodes and an MR-compatible amplifier system that transmits the amplified EEG outside the scanner. The patient must be as immobile as possible during the session. The magnetic gradient of the MR scanner induces large artifacts in the EEG. After the scanning session is finished, the artifacts will be removed by software to retrieve an EEG of reasonable quality (to retrieve a “clean” EEG); consequently, it allows us to mark the time of epileptic events. The next step is to build a mathematical model of what the BOLD signal should be at the voxels involved in the event. Voxels that took part in the event should have changes in their time courses as a result of each event in a predictable manner (in concordance with the HRF). Finally, the time course of every voxel is analyzed, and the voxels that have a correlative time course with the model are identified. Such voxels are either involved in the generation of the marked epileptic events in the EEG or a consequence of the event (3–6, 8–11).

Although the simultaneous recording of EEG–fMRI is one of the most valuable non-invasive tools for studying brain activity, it remains challenging to reach a high-quality signal of EEG and fMRI recorded simultaneously. Generally, simultaneous EEG–fMRI data are affected by various confounding factors and artifacts. The most important effective factor on the quality of the EEG and MRI recording is the immobilization of the head and electrode wires that can be reached by a plastic bag full of small polystyrene spheres. Besides, MRI-compatible sandbags are well-suited to immobilize the electrode wires on the way to the amplifiers (13).

Among the various artifacts, the artifact of MR gradient switching and ballistocardiogram (BCG) remain the major challenges in simultaneous EEG–fMRI study that make the EEG signal hard to interpret. Removing the fMRI scanner artifact is essential for the successful EEG–fMRI analysis. On the other hand, the presence of the BCG artifact does not necessarily lead to a complete failure in identifying epileptic events (13–16). Yet, eliminating the BCG artifact improves the readability of the EEG and is useful for detecting subtle events like small epileptic discharges (13, 17).

To reduce the MR artifacts, one of the effective ways is the blind source extraction (BSE) algorithm followed by the averaging-and-subtraction method (18). Also, Amini et al. (19) proposed an approach based on generalized eigenvalue decomposition (GEVD) and median filtering, which demonstrated a considerable improvement in reducing MR artifacts compared to the conventional methods.

For eliminating BCG artifacts, two well-known methods are independent component analysis (ICA) and principal component analysis (PCA) which keep the spikes intact. However, ICA usually makes a better distinction between artifact and non-artifact components and performs stronger in artifact removal while preserving the spikes (13). Also, for a significant number of events, the subtraction filter is better than the Fourier filter in producing distortion but impairs the readability of EEG because of leaving large remaining artifacts inside the frames (13). Another approach for BCG artifact correction is multiple-source correction (MSC) (20). First, the source of IEDs is extracted from the EEG data collected outside the scanner to avoid the distortion of EEG data during the correction of BCG artifacts. Then, the topographies of the BCG artifacts defined based on the EEG data acquired inside the scanner are added to the alternative model of IED sources. The combined source model is applied inside the EEG data. Lastly, the artifact signal is subtracted from the EEG without considerable distortion of the IED topography. Compared with the traditional averaged artifact subtraction (AAS) method, the MSC approach has improved the ability of IED detection, especially when the BCG artifact is correlated and time-locked with the EEG signal produced by the focal brain activity of interest (20).

In the study of Körbl et al. (21), 18 patients with epilepsy were studied with the common methods of BCG removal and the conventional method of using marked IEDs to perform event-related analysis. Besides, nine patients used the moiré phase tracking (MPT) marker to discard suspicious IEDs synchronous with the BCG before the event-related analysis. The results demonstrated no significant difference between the two groups. However, the IED timing distribution was significantly related to the cardiac cycle in 11 of the 18 patients recorded without the MPT marker, but only two of the nine patients with the marker. In patients recorded without the marker, failing to discard suspicious IEDs led to more distant activations and more inaccurate fMRI maps.

**Figure 2:**
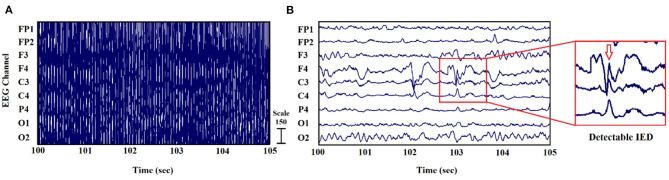
EEG signal recorded inside the MR scanner: **(A)** before and **(B)** after the elimination of gradient and BCG artifacts (24).

In some of our previous works (22–24), the MRI gradient switching artifact was removed by using the fMRIb algorithm (https://fsl.fmrib.ox.ac.uk/eeglab/fmribplugin), which first increases the sampling rate to 20 kHz and then applies a low-pass filter at 60 Hz. The fMRIb toolbox also removed the BCG artifact associated with cardiac pulsations. Figure 2 shows the EEG signals inside the scanner before and after the artifact removal procedure.

One of the other factors that affect the quality of the BOLD images is the signal loss due to variations in magnetic susceptibility, which alters the local magnetic field experienced by the subject's brain. For reducing this signal loss and increasing the ability to detect significant regions of BOLD signal changes, z-shimming is a practical technique. However, the question is whether this signal loss will be a limiting factor to identify the spike-related BOLD signal changes in patients with epilepsy. To find the actual effect of z-shimming in the results of identifying the spike-related BOLD responses, Bagshaw et al. (25) designed an experiment in which eight patients with temporal lobe epilepsy (TLE) underwent an EEG–fMRI session, and z-shimming was applied to their BOLD images. After comparing the intensities between z-shimmed and standard images and creating BOLD activation maps from the two sets of functional images using the times of spikes extracted from the EEG, it was found that the mean signal of the temporal lobes (TLs) increased 45.9 ± 4.5% as a result of z-shimming. Also, the percentage of the TL voxels above the brain intensity threshold increased from 66.1 ± 7.6% to 77.6 ± 5.7%. However, this increase in the signal did not make any significant differences in the statistical maps. So, the signal loss is not a limiting factor for identifying the spike-related BOLD responses in patients with TLE.

The magnetic field strength of the MRI scanners could be an effective factor for the reproducibility of the EEG–fMRI results, which makes the results reliable as a clinically valuable method. This issue was addressed by Gholipour et al. (26). Fifteen epilepsy patients, including seven who had one 1.5T and one 3T EEG–fMRI scans and eight who had two 3T EEG–fMRI scans were studied. Then, the IED-related BOLD responses acquired from equal numbers of the IED events were compared between the scans of each patient. In four of the 15 patients, the results of the comparison between two sets of scans acquired from 1.5T and 3T scanners showed more significant responses in 3T scans just because of the higher magnetic field strength. Also, for the eight patients, the results of comparisons between two consecutive 3T scans showed reproducible responses in five cases with similarity in the visual pattern of activation and partly differences in terms of maximum *t*-score and cluster size in some cases.

Reduction of motion interference has been considered in some studies. In a study of Klovatch-Podlipsky et al. (27), a method based on MR-compatible dual-array EEG (daEEG) was proposed to reduce the motion interference in the EEG–fMRI recordings. The EEG electrodes were organized into two sets of nearly orthogonally intersecting wire bundles, and virtual bipolar measurements were obtained both along and across the bundles. By applying ICA on the EEG data and using the fact that only motion interference is influenced by the cable orientation and is more prominent in across-bundle measurements, daEEG allows suppression of both BCG and non-BCG interference from the data. Testing this method in 10 patients with epilepsy and comparing the results with those of the Optimal Basis Set (OBS) (28–30) showed more detected spikes after using daEEG than after OBS in nine of the 10 patients.

In the GLM analysis, settings and preprocesses are also important for the localization of the epileptic sources and can be optimized. For instance, considering some video-EEG physiological confounds like eye blinks and swallowing as additional regressors can reveal further IED-related BOLD clusters which might be part of the epileptic networks (31). Mikl et al. in (32) used the EEG–fMRI data of 13 patients with pharmacoresistant epilepsy and an excellent surgical outcome and performed 240 statistical analyses for each patient including all possible combinations of the used preprocessing and GLM settings. The results showed that preprocessing type, i.e., mainly the basic pipeline, or cardiac artifact correction does not affect GLM-based analysis results. The IED stimulation time course shifted 2 s earlier than positions from the EEG description, and also the massive filtering of artifact (24 movement regressors, signals from white matter and CSF, and global signal) are considered as the optimal preprocessing pipeline. Also, they reported that the canonical HRF as the basis function led to the best results of GLM analysis in agreement with some previous studies like (33, 34). However, its superiority over more flexible basis functions may be due to the used concordance measure. It is noticeable that in another study, Lemieux et al. (35) used a more flexible model of the event-related response, a Fourier basis set, to identify regions of activation corresponding to non-canonical responses associated with individual IED in 30 experiments of patients with focal epilepsy. They reported that non-canonical activations were almost always remote from the presumed generator of epileptiform activity. Thus, the BOLD response to IED is primarily canonical and the non-canonical responses may represent a number of phenomena, including artifacts and propagated epileptiform activity.

### HRF and Spike Characteristics

In the common methods of EEG–fMRI analysis, a particular HRF is usually used for all patients. For example, the GLM framework models a prior knowledge of hemodynamic response in the design matrix and then explains the measured data by parameter estimation (10, 36–38). However, the real BOLD response to IEDs for each patient can be significantly different from the healthy controls (6, 39, 40). Even in a specific patient, the shape of HRF varies with different brain areas and also is time-variant in each area (41, 42). The delay of the estimated function in a patient is different from those of the common theoretical models (43, 44). Using patient-specific HRF increases the detection sensitivity of epileptic spikes in EEG–fMRI (40). For instance, van Houdt et al. (17) used a finite impulse response approach for estimating the HRF from a dataset including 42 IED sets acquired in 29 patients and observed that more brain regions were active consistent with the EEG focus compared to the classical approach supposing a fixed HRF for each voxel in the brain (26 vs. 16).

Using multiple HRFs with peaks ranging from 3 to 9 s increases the BOLD response compared with using the standard HRF alone (11, 45). It was shown that the standard HRF that peaked at 5.4 s was more proper in detecting positive BOLD responses, and the HRFs that peaked later than the standard were more accurate for negative BOLD responses (45).

It has been observed that the results of EEG–fMRI analysis are influenced by the evaluation of the EEG signal and the scanning techniques more than the HRF model. Thus, although the HRF model influences the results of EEG–fMRI analysis, it may not be the main parameter in clinical practice (46).

Regarding the epileptic spikes in the EEG signal, it is revealed that the activation in BOLD response from EEG–fMRI analysis depends on the number of IEDs occurring during data acquisition (11, 17). However, the spiking rate is not the only influencing factor in the presence of the BOLD response. BOLD responses were seen in patients who had very few spikes, and a lack of response was noted in patients who had a high spiking rate (11).

**Figure 3:**
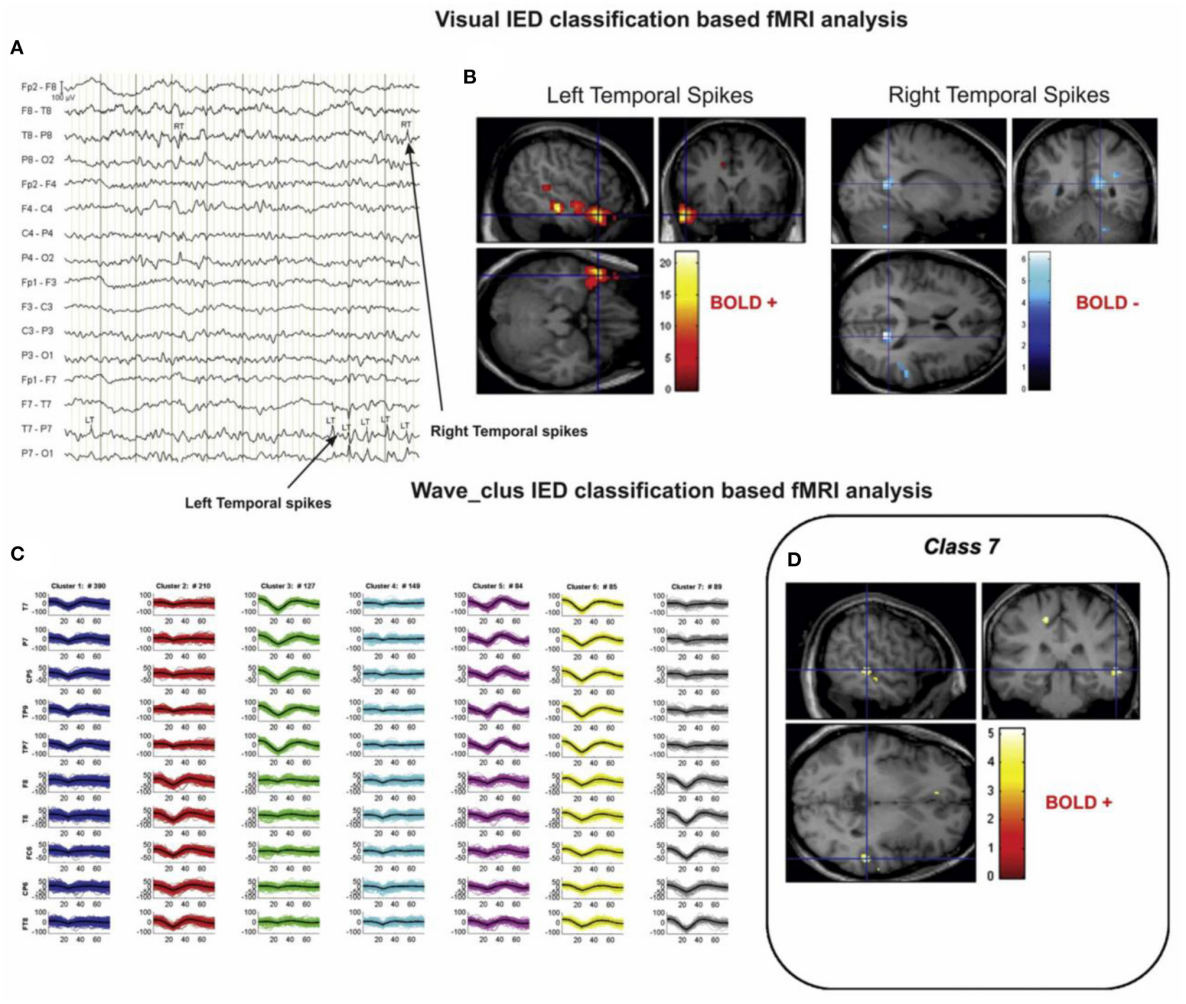
A sample of visual and algorithmic classification of the IEDs. **(A)** The result of visual classification from the bipolar montage (64 channels) of EEG recorded inside the scanner is performed by an expert. **(B)** The results of EEG–fMRI analysis, based on visual-IED labeling. **(C)** The seven classes identified using the algorithmic classification. **(D)** The result of EEG–fMRI analysis, associated with class 7 of identified IEDs. All the fMRI results are overlaid on the subject's T1-weighted image (48).

Another issue is the spike identification that can be done automatically (20, 47) or by an expert. According to the study of Pedreira et al. (48), the automated spike-sorting algorithms for the classification of IEDs increase the value of EEG–fMRI analysis and mapping of IED-related BOLD responses (Figure 3). However, there is uncertainty in the results of spike identification because of the false detections and missed events. Huiskamp et al. (49) evaluated the impact of these two errors on the significance of the expected fMRI activation and revealed that the effect of missed events is larger in deteriorating the expected results. According to this study, although the uncertain spikes cause errors in IED-related BOLD responses, if they are considered as the events and included in the analysis, the responses will be closer to the expected results.

In the same direction, the aim of Flanagan et al. (50) was to find the influence of inexact or unreliable marking of EEG epileptiform events on the result of statistical parametric mapping (SPM) analysis in EEG–fMRI studies of patients with epilepsy. In this paper, the EEG–fMRI data of 10 patients with epilepsy were analyzed, and epileptiform events were marked. Then, the effect of omitting, mislabeling, and inconsistent timing of events was observed separately, considering the numbers of voxels above the threshold in the resulting SPM analysis. The results showed that omitting true epileptiform events decreased the number of above-threshold voxels. Mixing epileptiform and non-epileptiform events usually (but not always) caused a similar decrease. Inconsistent timing of events for small (<200 ms) and large (>500 ms) inconsistencies had small and large effects on the results, respectively. This suggests that accurate marking up of epileptiform events in EEG is still one of the most important factors for obtaining reliable results from EEG–fMRI analysis.

Besides, multiple fast fMRI sequences have been recently developed, one of which is magnetic resonance encephalography (MREG). Comparing MREG with the traditional sequence of echo-planar imaging (EPI) has revealed that MREG gives higher maximum *t*-values than EPI (49). However, Safi-Harb et al. in (51) reported that EPI yielded a better true positive rate and larger cluster size than MREG using a proper threshold. Also, it was shown that the HRF shape had a larger effect on MREG detection than EPI. Additional studies are needed to make a definitive judgment on their relative sensitivity. In terms of localizing the epileptic network, Jäger et al. (52) state that high-density EEG and fast fMRI seem to improve EEG–fMRI analysis results.

### Pre-spike BOLD Signal Changes

**Figure 4:**
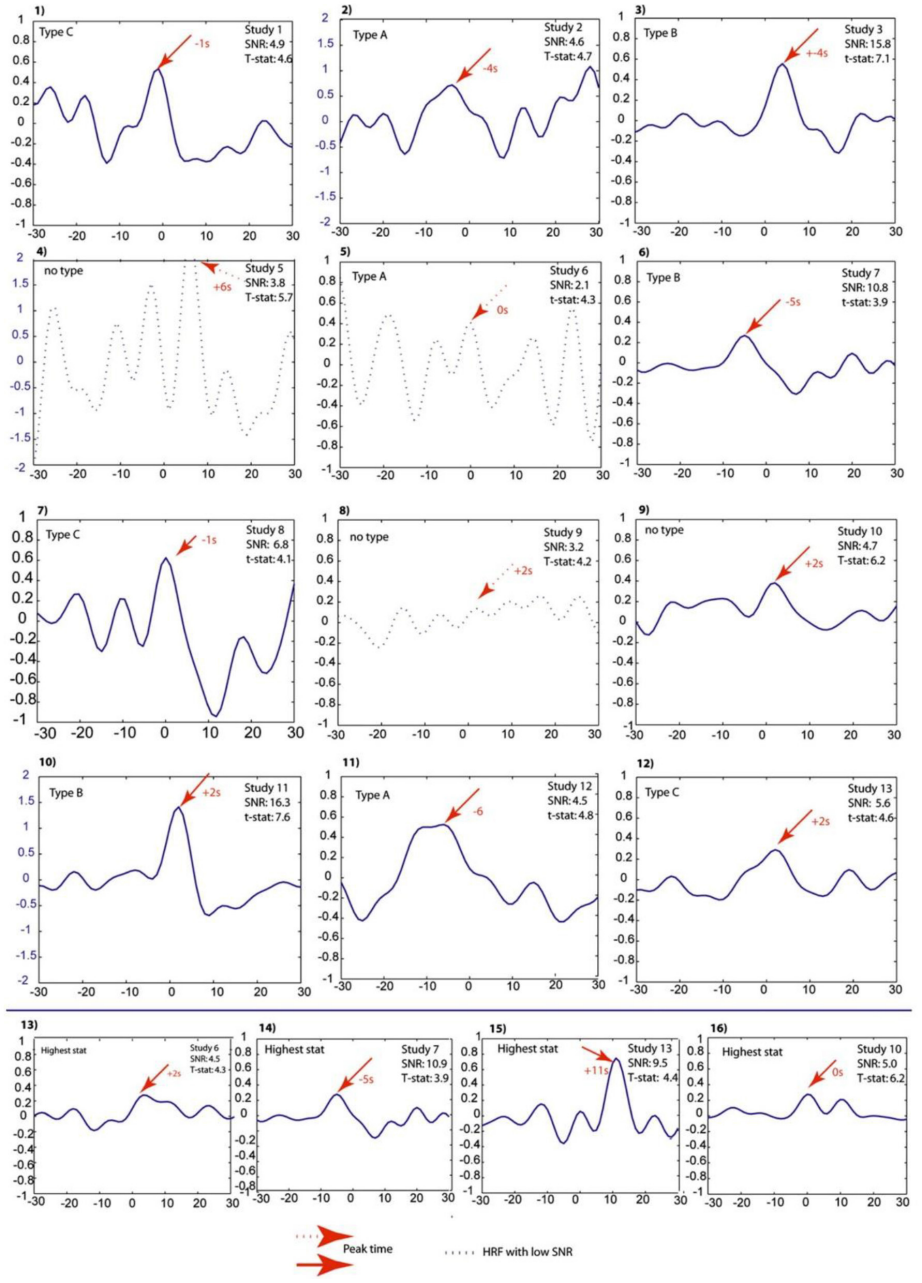
Projected HRFs illustrated according to a sequence of patients. The bottom row shows HRFs calculated over the area with the highest t-statistic. The HRFs which presented with dotted lines are those that did not pass the SNR criterion of 4.5. The Blue scales had to be adjusted to obtain visibility of the HRF and thus differ from the rest. The HRF shape differences and their sporadic early peak times are obvious (54).

Hemodynamic changes that are time-locked to spikes may reflect the propagation of neuronal activity from a focus, or conversely the activation of a network linked to spike generation (53). That is why pre-spike concordant BOLD signal changes may contain information about the epileptic networks. In a study of Jacobs et al. (54), five patients with idiopathic focal epilepsy and six patients with symptomatic focal epilepsy were studied. Spike timing was identified, and HRFs were calculated as the most focal BOLD response to model the regressors of statistical analysis with the timing of spike events convolved to HRFs peaking at −9 to +9 s around the spike. The results showed pre-spike BOLD responses in 11 of the 13 studies which were more focal and related to the spike field than post-spike responses (Figure 4).

The question of whether these pre-spike BOLD responses were the result of a synchronized neuronal discharge was yet to be investigated. In another study (55), four patients with pharmacoresistant focal epilepsy were selected by showing both pre- and post-spike BOLD responses concordant with the EEG focus during the session of EEG–fMRI recording. Then, they underwent stereo-EEG (SEEG) as part of their pre-surgical evaluation to specify the origin of pre-spike BOLD signal changes. Pre-spike BOLD signal changes in the spike field area were analyzed using HRFs with peaks ranging from −9 to +9 s around the spike. After that, SEEG signals were analyzed for detecting electrographic changes consistent with the time and location of the early HRF responses. The results showed that only one of the patients had a consistent SEEG interictal discharge. No electrographic changes were detected in the rest of the patients, consistent with the early HRF responses in period and location. Therefore, the early BOLD signal change usually reflects a metabolic event that does not seems to be the result of a synchronized neuronal discharge.

### BOLD Response to IEDs

There may be a direct relationship between the BOLD signal changes and overall synaptic activity (56, 57). The generation mechanisms of interictal discharges are unknown in humans, but the cortical development abnormalities have distinctive interictal discharges (56). For a reliable localization of epileptic foci using EEG–fMRI, we need IEDs correlated with the BOLD signals recorded simultaneously. However, the epileptogenic regions with correlated signals have not yet been thoroughly understood (58). Thus, a considerable part of the literature is centered on the behavior of BOLD signal changes associated with interictal discharges.

Federico et al. (56) focused on the BOLD signal changes associated with interictal discharges in six patients with malformations of cortical development and seizures using spike-triggered fMRI 3T. They revealed four positive changes in the lesion and four negative changes surrounding the lesion, five changes at distant cortical sites, and three subcortical sites (basal ganglia, reticular formation, or thalamic). Waites et al. (33) studied two patients with frequent epileptiform events and concluded that interictal discharges result in BOLD responses distinctly different from those obtained by examining random events. Besides, Bonaventura et al. (59) investigated BOLD responses related to epileptic EEG abnormalities in 31 partial and 12 generalized epilepsy patients and revealed that there are obvious associations between BOLD results and EEG abnormalities in 21 cases with 18 concordant to electro-clinical findings.

Another strand of literature focuses on idiopathic generalized epilepsy (IGE). For instance, Briellmann et al. in (60) analyzed the data from 17 patients with IGE and frequent, stereotypical generalized discharges that were present in 14 of them during scanning. As reported, the cortical changes were found in all patients, and subcortical changes were found in only seven of the patients who had bursts of rhythmic discharges during scanning. Fifty-five percent of the patients showed deactivation in the posterior cingulate, and two of the patients who had marked activation and electro-clinical absences during scanning showed thalamic signal change.

Tyvaert et al. (61) analyzed the EEG–fMRI data from 10 patients with IGE during generalized spike-and-wave discharges (GSWDs). The HRFs were calculated in four ROIs related to the left and right thalamic structures and were compared within and between them. The results pointed to an activation of the centromedian and parafascicular (CM-Pf) nuclei and then of the anterior nucleus during GSWDs. This suggests that the early propagation and maintenance of epileptic discharges may belong to the posterior intralaminar nuclei and anterior nucleus, respectively.

In another study (62), the EEG–fMRI data of 83 patients with medication-refractory IGE (R-IGE) were analyzed, and statistical parametric maps concerning the BOLD response were generated. Thirty-six patients were identified as cases with absence seizures. It was inferred that when thalamic BOLD changes peaked at ~6 s after the onset of absence seizures, the other areas, including the prefrontal and dorsolateral cortices, showed brief and non-sustained peaks at ~2 s earlier than the thalamic peak. Also, TL peaks occurred at the same time as the thalamic peak, with a cerebellar peak occurring ~1 s later. Thus, the origin of absence seizures may be the widespread cortical (frontal and parietal) regions and sustained in subcortical (thalamic) areas, representing the cortical onset of epileptic seizures with propagation to the thalamus.

In a study of Benuzzi et al. (63), 18 patients with IGE and absence seizure (AS) were studied, and the event-related analysis was performed using the onset and duration of GSWD as one regressor and GSWD offset as another. The results pointed to a thalamic activation and a deactivation in pre-cuneus/posterior cingulate related to the GSWD onset and a BOLD signal decrease over the bilateral dorsolateral frontal cortex GSWD termination.

For a 28-year-old focal epilepsy patient with left frontal seizures who were treated with oxcarbazepine (1,200 mg/d), two sessions 1 month apart of continuous EEG–fMRI with two different runs for each session were held. The IEDs were extracted using the location of the electrodes with the maximum amplitude of the epileptiform activity, and the colocalization of fMRI clusters was established based on the anatomical lesion and IEDs. In both runs of the first session, a unique left frontal main cluster was identified in the left opercular region colocalized to IEDs and near the posttraumatic lesion. However, in the second session, two main clusters were detected in the inferior frontal gyrus of both hemispheres. Therefore, EEG activity did not considerably change within each session, whereas the spatial distribution of interictal events showed significant variations between the sessions (64).

In another study, Flanagan et al. (65) reviewed the EEG–fMRI data of 27 patients with focal epilepsy in terms of the location and extent of the IEDs and the resulting pattern of significant BOLD responses. This study characterized important features of the BOLD responses associated with the IEDs and confirmed that the piriform cortex is a common node underlying IEDs and suggests a purpose for further study and potential therapy.

Fahoum et al. (66) studied 32 patients with TLE, 14 patients with frontal lobe epilepsy (FLE), and 20 patients with posterior quadrant epilepsy (PQE) and acquired the patterns of cortical and subcortical BOLD responses related to focal IEDs using a group analysis. The patients with TLE showed activations in the midcingulate gyri bilaterally, ipsilateral mesial and neocortical temporal regions, insula, and cerebellar cortex, and also the most widespread deactivations in the default mode network (DMN) areas. The patients with FLE showed activations in the midcingulate gyri bilaterally, ipsilateral frontal operculum, thalamus, internal capsule, and the contralateral cerebellum, and also deactivations in the DMN areas. Lastly, the patients with PQE showed only deactivations in the DMN area.

### Negative BOLD Signals

For the cases of negative BOLD signals, the epileptogenic regions with correlated signals are not also completely understood (58). For explaining the negative BOLD signals, three different scenarios could be as follows: An overcompensating cerebral blood flow decrease could accompany a decreased metabolism as a “normal” negative BOLD response; the epileptic activity could produce an increased metabolism without adequate blood flow change resulting in a negative BOLD effect; and the oxygen consumption could stay constant throughout the IED event while at the same time, a reduced local blood flow is induced (58).

The study of Rathakrishnan et al. (67) aimed to explain the negative BOLD responses seen in the source of epileptiform discharges by the undershoot of an antecedent positive response. In analyzing the EEG–fMRI data of 82 patients with focal epilepsy, only eight patients showed a focal negative BOLD response in the spike field area using models with HRFs peaking from −9 to +9 s around the spike. Thus, the origin of negative BOLD responses in the epileptic foci is not an initial positive BOLD response and remains unexplained in most patients.

To determine the origin of BOLD negative response to the IEDs, Pittau et al. (68) studied two groups of patients, each including 15 patients with significant positive and negative BOLD responses within the IED region, and explored the relationship between the type of response (activation/deactivation) and several IED characteristics. The results denoted that the IEDs of patients with deactivation were more frequently of long duration with larger involved cortical areas and more focused in the posterior quadrant. Also, the IEDs accompanied by a slow wave were present in 87% of the deactivation group and only in 33% of the activation group which is the critical feature reliable for focal deactivations.

## Localization Approaches

### Localization of Epileptic Focus Using EEG–fMRI

The EEG–fMRI analysis has been widely used for the localization of epileptic foci. However, the respective approaches need more refinement to be reliable for pre-surgical decision making (69). In the following section, the results of applying various analysis methods are reported.

#### Conventional Analysis

Simultaneous EEG and fMRI recordings can reveal the source of spiking activity that is highly correlated with epileptic foci and epileptogenic lesions in a large number of patients. However, many of the patients have no significant activation for unknown reasons (12). In the study of Al-Asmi et al. (12), the EEG–fMRI data of 38 patients with focal epilepsy and frequent spikes were analyzed in terms of fMRI activation using two methods: (1) the significance of the t-statistic value at every single voxel and (2) the significance in the clusters of contiguous voxels based on random field theory (70). The concordance between the spike location of EEG and anatomic abnormalities of MRI and other EEG and clinical measures were taken into consideration. From the analyzable ones, activation regions were obtained in 39% that were concordant with EEG source localization in nearly all of them. Forty percent showed activation without any MRI lesion, and 37.5% showed activation near or inside the lesion.

In a study of Zijlmans et al. (71), the EEG–fMRI data of 29 patients with epilepsy were studied, and 46 sets of IEDs were identified in the agreement between two experts. The BOLD response related to each type of IEDs was modeled in an event-related design using a canonical HRF with a temporal derivative, and statistical maps of activity were created. The results showed an improvement in the localization of epileptic focus and opened new prospects for surgery. For instance, at least one significant positive BOLD response topographically concordant with the IEDs was found in eight patients who were rejected for surgery due to reasons like unclear focus or multifocality. This is, therefore, a valuable tool in the pre-surgical evaluation of patients with epilepsy.

Besides, in the study of De Tiège et al. (72), the IEDs were extracted and segregated into separate regressors applying a half-maximum amplitude cutoff in six children with pharmacoresistant focal epilepsy. The regressors were then convolved with the canonical HRF and its temporal derivative for an event-related fMRI analysis. The results showed significant activations in four children, colocalized with the presumed epileptic focus, activation and deactivation in one child, and a widespread deactivation in another.

In another study, Grova et al. (73) evaluated the level of consistency between EEG source localization and BOLD responses using two comparison strategies: (1) MEM concordance, which is the comparison between EEG sources detected using Maximum Entropy on the Mean (MEM) and fMRI clusters of significant BOLD response and (2) fMRI relevance: if sources located in an fMRI cluster could explain some scalp EEG data, the assessment of the fMRI-relevance index α would measure when this fMRI cluster was used to constrain the EEG inverse problem. For this purpose, seven patients with focal epilepsy underwent EEG–fMRI and an EEG recording outside the scanner. The results of combining two mentioned strategies to report the concordance between BOLD response and EEG sources showed that from 62 fMRI clusters assessed by standard event-related analysis, 15 were highly concordant with EEG according to both strategies, five were concordant only according to the fMRI-relevance index, 30 were not concordant, and 10 clusters had a significantly negative α index suggesting EEG–fMRI discordance.

Avesani et al. (74) analyzed the EEG–fMRI data of a patient with symptomatic epilepsy to find the linkage between the “epileptogenic” zone and the “irritative” zone, which is the meticulous cortical distribution of spikes. They used EEG signals as paradigms in the fMRI study and compared the EEG interictal slow-spiked wave with the normal EEG conditions. The results showed a BOLD signal increase around the epileptogenic area in the left neocortical temporal region, laterally and posteriorly to the porencephalic cavity, representing a connection between “epileptogenic” and “irritative” areas.

In a study of Jackson (75), Jackson extracted 46 IED sets from 29 patients with epilepsy who were excluded for surgery on unclear foci. Also, he analyzed the fMRI data to identify BOLD, significant responses, and topographical concordance with IEDs. Fifteen patients showed significant positive or negative BOLD responses. Eight patients showed IED-related positive BOLD responses. Four of the five patients with presumed multifocality showed multiple epileptic foci. Four of six patients with unclear foci showed a confined focus, opening new predictions for surgery.

Besides, in the study of Liu et al. (76), the EEG–fMRI analysis for the localization of partial epilepsy includes extracting and convolving the spike times with a two gamma-variate canonical HRF and adding the result as a task regressor to the SPM design matrix. This approach was applied to the data of eight EEG–fMRI sessions acquired from six patients with partial epilepsy and showed six with activation and deactivation, one with activation only, and one with deactivation only. Seven of the observations corresponded to electroclinical localization of epileptic focus. As reported in this study, the concordance seems to be more associated with positive BOLD responses, and the response to deactivation seems less associated with IEDs. Such studies generally demonstrate that IEDs may be revealed in the brain regions well beyond the presumed area in which they are generated (77). In the study of Moeller et al. (78), the EEG–fMRI data was acquired from nine patients with non-lesional frontal lobe epilepsy (FLE). Using four HRFs, IED-related BOLD responses were obtained and compared to the spike topography determined by BESA as a voltage activation map. The results showed a concordance between the positive BOLD response and the spike localization in eight of nine patients.

Borelli et al. in (79) studied a patient of focal cryptogenic epilepsy with speech arrest seizures and bilateral synchronous spike and wave scalp EEG pattern (secondary bilateral synchrony). Following the conventional analysis of EEG–fMRI data, the IEDs were identified, convolved with a two-gamma canonical HRF, and added to a single-subject GLM. The statistical map of significantly activated voxels showed an explicit BOLD response over the left supplementary motor area (SMA) and, to a lesser degree, over the homolateral motor strip. Forty-three patients with focal epilepsy were studied in (80), and BOLD responses associated with IEDs, including at least five significant contiguous voxels, were extracted and labeled as consistent and inconsistent with the EEG spike field and contributory or not contributory, based on whether or not they provided additional information to EEG about the epileptic foci. The main analysis included convolving a regressor developed using the time and duration of each IED-type event with four HRF peaking at 3, 5, 7, and 9 s and adding all the regressors to GLM. Thirty-three patients who had more than two IEDs during recording were shown to have significant BOLD changes, among which 29 were considered consistent, and 21 were contributory. The BOLD responses were validated in 12 of 14 patients having intracerebral EEG or a focal lesion on MRI.

Ten patients with atypical benign partial epilepsy (ABPE) underwent simultaneous EEG–fMRI, and several types of IEDs were extracted from their data in (81). The analysis of BOLD signal changes associated with each IED type showed distant significant responses in cortical and subcortical structures for 31 cases out of 33 among which 21 were concordant with the spike field. Also, to find the responses across the patients, group analysis was performed and showed a thalamic activation. It is noteworthy that the revealed activation in ABPE was analogous to the outlines showed in studies of rolandic epilepsy and continuous spike-wave during slow sleep (CSWS). Zhang et al. (82) investigated the results of pre-surgical EEG–fMRI analysis and iEEG monitoring in a patient with seizure recurrence after epilepsy surgery. They suggested that EEG–fMRI is a useful tool for pre-surgical evaluation but requires caution. Also, the intact seizure foci in the remaining brain may cause the non-seizure-free outcome.

In previous studies of improvement in the localization of epileptic foci, Tousseyn et al. (83) used the conventional GLM-based approach for the localization of epileptic focus in a semi-automated manner by proposing a spike identification method as an alternative for the challenging and time-consuming visual spike detection. In this method, a patient-specific spike template was generated by averaging the spikes observed on the EEG outside the scanner, and the cross-correlations were calculated between the template and the EEG inside the scanner. Then, the result was binarized by a threshold determined from healthy controls and convolved with a canonical HRF to be used as the regressor of GLM. Examining this semi-automatic method on the EEG–fMRI data of 21 patients with refractory focal epilepsy yielded a good performance with the optimal area under the ROC curve of 0.77.

Sandhya et al. (84) studied three patients with drug-resistant reflex epilepsy, including eating, startle myoclonus, and hot water epilepsy using conventional analysis. The results showed frontoparietal network activation pattern in the patient with startle myoclonus epilepsy concordant with SPECT, fronto-temporo-parietal involvement in the patient with eating epilepsy concordant with SPECT, and fronto-parietal-occipital involvement in the patient with hot water epilepsy. In research conducted by Tousseyn et al. (85), 28 patients with refractory focal epilepsy underwent EEG–fMRI and subtraction ictal SPECT co-registered to MRI (SISCOM). Comparing the perfusion changes during seizures obtained from SISCOM and spike-related BOLD signal changes obtained from EEG–fMRI revealed a concordance between the BOLD responses and EEG spikes in 27 cases, a significant spatial overlap between hyperperfusion on SISCOM and hemodynamic changes on EEG–fMRI in 20 cases, and significant overlay between ictal hypoperfusion and interictal deactivation in 22 cases.

#### Dipole-Based Analysis

The spike source reconstruction of EEG is generally consistent with the BOLD localization (86). It can therefore be used for the localization of epileptic focus. Some of the source localization methods are fixed dipoles, moving dipoles, LCMV (linearly constrained minimum variance), spatial filtering, MUSIC (multiple-signal classification) dipole scans, and LORETA (low-resolution tomography) (87).

Lemieux et al. (86) recorded a 12-channel EEG inside a 1.5T MRI scanner in six epilepsy patients with partial seizures. A T1-weight volume scan and a 64-channel scalp EEG outside the scanner were obtained from each patient. Having extracted spikes from the EEG signals, they performed the source reconstruction using three generator models consisting of multiple moving dipoles, MUSIC dipole scan, and current density reconstruction (Curry 3.0 software) to localize spike generators and compared its results with the spike-triggered fMRI activation maps (SPM96 software). They concluded that the spike generator was located inside or in the same fMRI activation lobe. Therefore, source reconstruction was generally consistent in EEG generator models and fMRI individual clusters.

Bagshaw et al. (88) showed that EEG–fMRI results should not constrain MEG and EEG inverse solutions for equivalent current dipole approaches in epilepsy and that the use of distributed source modeling would be a more appropriate way of combining EEG–fMRI results with source localization techniques. They analyzed the EEG–fMRI data from 17 patients with focal epilepsy and compared the results of spatiotemporal dipole modeling with the peak and closest EEG–fMRI activations and deactivations. They reported that, generally, the distance from the dipoles to the voxel with the highest positive *t*-value and nearest activated voxel was 58.5 and 32.5 mm, respectively, and also that for deactivations was 60.8 and 34.0 mm, respectively. It is obvious that these values are significantly higher than what is generally observed with ERPs, possibly due to a comparatively broad field that could lead to deep artificial dipoles and also the prevalence of EEG–fMRI responses away from the focus of the epileptic activity hypothesis.

Recently, a new method has been proposed for measuring the physical distance between the BOLD clusters and selected component dipoles to improve the identification of epilepsy-related components in the EEG–fMRI analysis (22).

In a study of Secca et al. (89), two patients with idiopathic occipital lobe epilepsy (OLE) were studied in terms of the source analysis using instantaneous regional dipoles at the peak of averaged detected spikes with a three-layer boundary element model (BEM) of volume conduction. Relating the BOLD effect with interictal spikes using a standard Gamma HRF with derivatives, the authors were able to detect BOLD clusters and compared them with the malformative lesion and diagnosed seizure symptomatology, which was moving the right hand, which yielded a very good concordance for each patient between the BOLD clusters, malformative lesion, and the seizure symptomatology.

In another study (90), three patients with idiopathic childhood occipital lobe epilepsy (OLE) underwent EEG–fMRI. EEG source analysis was conducted using prompt moving dipoles at the peak of averaged spikes, which were detected visually, with a standard three-layer boundary element model (BEM). Next, the BOLD activation map was acquired coupled with the incidence of EEG spikes. The results showed no changes in the BOLD activation in the cortex adjoining to the source analysis dipoles. Deactivation analysis showed several clusters with more consistency to the localization of EEG source analysis over the right parietal area. Therefore, the spatial overlap between EEG source analysis results and the BOLD activation map was not quite acceptable. However, the fMRI results were more consistent with the clinical advents.

**Figure 5:**
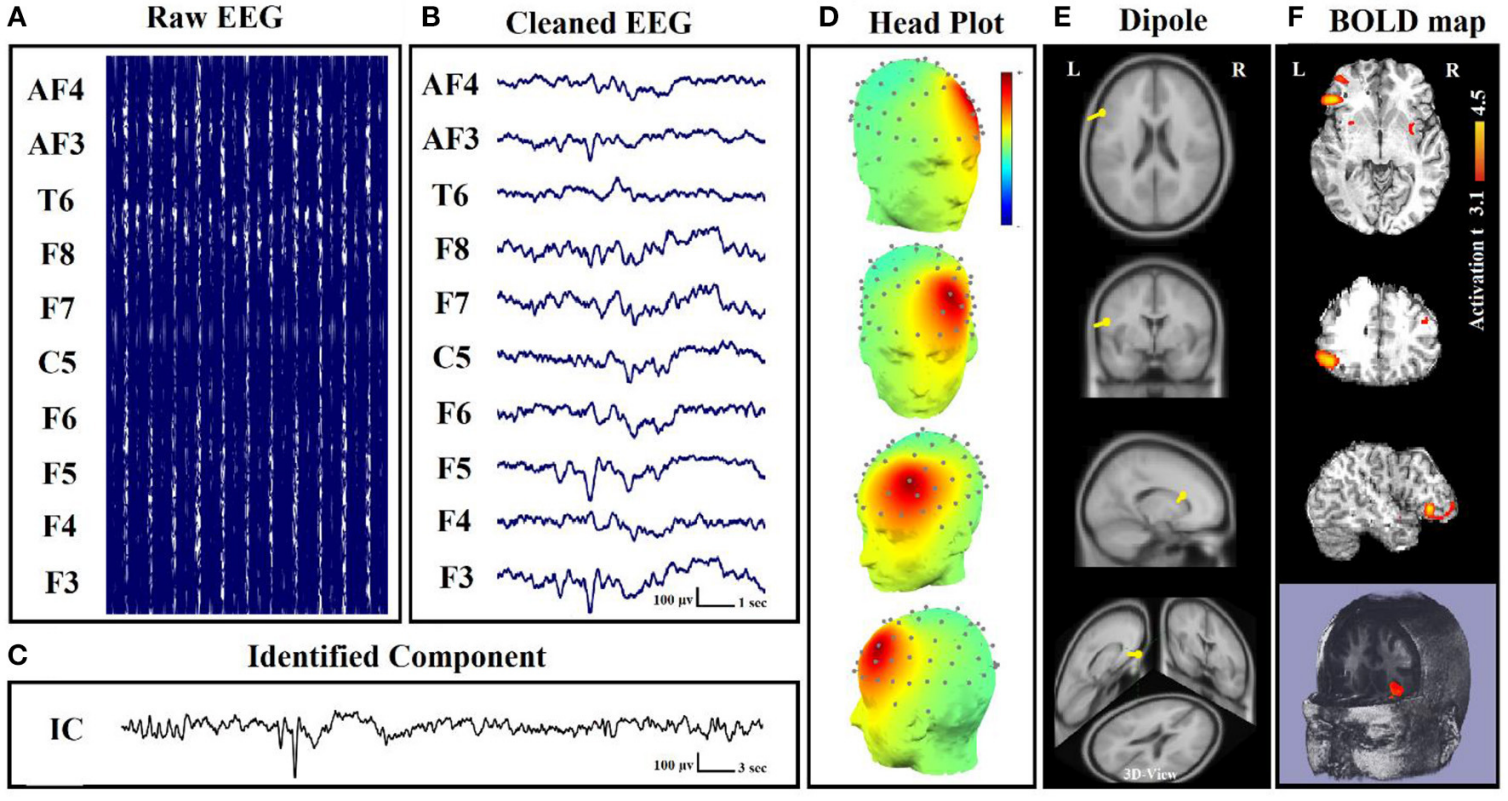
Dipole-related BOLD response showed a focal activation in the left frontal lobe. **(A)** Raw EEG data acquired inside the MR scanner. **(B)** Cleaned EEG after removing the gradient artifact. **(C)** Identified component time series. **(D)** The component identified on scalp EEG located in the left lateral frontal lobe. The active area is marked by yellow-red color. **(E)** Dipole localization of the identified generator in deep brain structures. **(F)** Localization of the generator applying simultaneous analysis of EEG–fMRI (22).

In our works, we used a dipole-based method for the evaluation of our localization method (22). This study revealed that BOLD responses were related to epileptic spikes in various brain regions of patients with refractory focal epilepsy (Figure 5). So, dipole-based analysis can help in the localization of epileptic focus in patients with focal epilepsy and is comprised as part of the pre-surgical evaluation for patients with pharmacoresistant epilepsy.

#### Component-Involved Analysis

Besides the SPM that is a hypothesis-driven method, ICA is a data-driven method (91) that can be used to find independent components of epileptic sources and add them to the simultaneous EEG–fMRI analysis. The component-involved approaches can also corroborate a negative decision concerning surgical candidacy in some cases (24).

In the study of Penney et al. (92), the EEG–fMRI data of a patient with refractory right TLE were studied. Applying spatial ICA (sICA) to the BOLD fMRI measurements, a hemodynamic response model signal derived from the task-related spatial ICs and used as a regressor in SPM to generate the significant BOLD activity maps. The results of this approach were compared to the same results using a conventional regressor generated from a canonical hemodynamic model and revealed a concordance between the activated regions. So, this sICA-based model may improve the accuracy of localizing epileptic focus.

Also, in the study of Rodionov et al. (93), the findings of sICA compared to the EEG-based GLM analysis in eight patients with focal epilepsy. The spatiotemporal concordance was assessed between the BOLD-related ICs and GLM-derived results to find one IC related to IED-based GLM results. So, the remaining candidate BOLD-related ICs may include the IEDs which were not apparent on the EEG. So, the sICA-based approach can be used to recognize the SOZ and may be helpful when the epileptic activities are not evident on the EEG signal.

Sercheli et al. in (94) used the EEG dipole modeling analysis to ICA components for the localization of epileptic focus in a patient with right mesial TLE before and after a successful resecting of the epileptic region. With this aim, the same dipole source localization of ICs was performed within a three-shell boundary element model of MNI standard brain using DIPFIT2 plug-in of the EEGLAB toolbox. The conventional approach was also performed to evaluate the results of ICA dipole modeling analysis, which used the fMRI statistical analysis with a regressor of IEDs convolved to a gamma HRF. The results of the conventional analysis showed a right hippocampus induction of the large interictal activity in the left hemisphere. However, the results of dipole modeling analysis showed a widespread distribution of activity, and almost only a quarter of the dipoles were near the right hippocampus region. Using just the EEG analysis to precisely identify the epileptic sources is too weak even by a sophisticated method like ICA.

Marques et al. (4) suggested a technique based on the ICA and applied it to the EEG–fMRI data of nine patients with epilepsy. In this method, after using ICA on the EEG data, the candidate ICs were one or two components that were most powerfully related to IED activity considering only the signal, which is over three standard deviations from the mean of the respective channel. The candidate components were convolved with the canonical HRF and added as the regressor to GLM of the BOLD signals. The results of this method were compared with the conventional method and showed concordance in six patients with more significance and extent in most of them, compared to the conventional method results. The rest of the three patients showed no significant activation using the conventional method to be comparable.

In another study (95), various IED types were classified using ICA and temporal correlation of ICs with the raw EEG channel. Then, the time pulse of each IED type was convolved with a canonical HRF and added separately to GLM for finding the focus of each identified IED type. This method was used in 10 patients with epilepsy including two cases with unknown sources of activity using the conventional method. The results of the proposed method on two patients with unknown source of activity showed some foci consistent with electroclinical data, and those on the rest of the eight patients showed significant activity from at least one type of IED consistent with the conventional method that proves the efficacy of this method for the localization of epileptic focus.

In a study of LeVan (96), 15 patients with focal epilepsy underwent simultaneous EEG–fMRI, and ICA was applied to each of their fMRI data. Then, matching a canonical HRF to the ICs time series in the IEDs' time, the components associated with the seizures were found, and the matched HRFs were used to regulate the sign and delay of the actual HRF peaks. HRFs with an obvious peak were used to create the activation maps of significant BOLD signal changes and compared with the results of a common GLM method. Evaluating the concordance of results with the presumed epileptic foci determined by clinical history, EEG, and MRI abnormalities revealed that the ICA maps were correlated with the GLM maps for all the patients with an activation network that always included the presumed epileptic foci, but more widespread, as much as 20.3% of the brain volume averagely.

Besides, in the study of Leite et al. (97), five metrics including total power, un-normalized root mean square frequency, un-normalized mean frequency, root mean square frequency, and mean frequency were calculated and added to GLM using the performed ICA on the EEG data. For calculating these metrics, the power spectrum was acquired from time-frequency analysis using Morlet wavelets. The metrics were calculated for only the component spectrums presenting spectral alterations during the events identified by the neurophysiologist. In a practical case, applying this method to the EEG–fMRI data of one patient with epilepsy produced wider and more significant activation maps compared to the conventional method using a standard square waveform regressor. Furthermore, the EEG metrics with a frequency content were better predictors of the BOLD signal than global power metrics, supporting previous theoretical predictions and experimental evidence. This method was also tested in (98) for four patients with epilepsy and again revealed more significant activations compared to the conventional analysis.

In another experiment (99), a 10-year-old male patient with epilepsy underwent simultaneous EEG–fMRI for investigating the dynamic responses of epileptic networks. ICA was used in fMRI data, and IED-related ICs were detected fitting an HRF to their time courses at the time of the IED event. Then, the epileptic source of the EEG signals was identified by convolving a canonical HRF with the time pulse function of IEDs as a regressor of a GLM analysis. Comparing IED-related ICs with the EEG source imaging of IEDs in terms of HRF peak delay and spatial consistency using minimum norm estimation (MNE), the fMRI ICs were classified into spatially consistent and inconsistent ones. So, the spatially compatible ICs with early HRF peaks which resulted from spatial-temporal EEG–fMRI fusion (STEFF) would be the possible indicators of the epileptic focus.

Formaggio et al. in (100) presented a novel automatic approach for simultaneous EEG–fMRI to identify the epileptic focus based on ICA and wavelet analysis. This method consists of four steps: (1) applying ICA and selecting components related to IEDs based on their power using a wavelet time-frequency representation because of higher amplitude in IED activity than background activity and the non-stationarity of the signal; (2) eliminating unselected components and reconstructing the EEG signal with only the IED-related components; (3) calculating the cross-correlation between the reconstructed EEG and the original signal to compare and find the IED channel with the highest correlation coefficient, and also building the power signal using a partial maximum of the estimated time-frequency power spectrum of IED channel for each epoch of 3.7 s by wavelet analysis; and (4) convolving the power time series with the canonical two-gamma HRF as the regressor of GLM. After validating this method on simulated data and applying it on real EEG–fMRI data, including five patients with partial epilepsy and two normal subjects, the results showed an extension in current knowledge on epileptic focus localization and suggested that BOLD activation related to slow activity might contribute to the localization of epileptic foci even in the absence of clear interictal spikes.

Franchin et al. (101) presented a method to classify the ICs of fMRI using an elevated algorithm to distinguish the sources of interest from noisy signals. Applying this method for estimating the BOLD activations related to epilepsy and comparing its results with the conventional GLM approach showed that the activations resulted using this method comprised subareas of the those resulted from the conventional analysis, even with partial discordant patterns of the activated areas, and also consists of additional negative regions implicated in a default mode of brain activity, and not clearly identified by GLM.

In our previous study (24), we attempted to localize the focus of epileptic seizures by identifying the neural behavior of the seizures and detecting the related components as a regressor and the input of a GLM model. For this aim, 28 sets of IEDs from nine patients who were excluded for surgery because of unclear focus in four, presumed multifocality in three, and a combination condition in two cases were analyzed. The result of localization showed an improvement in localization of foci using the component-based approach, which includes five of six patients with unclear foci, advocating one of the foci in five patients with assumed multifocality, confirming multifocality in one of them, opening new prospects for surgery in seven of the patients. Also, in two of the patients, intracranial EEG supported the EEG–fMRI results.

In a study of Hunyadi et al. (102), the ICA was used in the fMRI time series collected from 28 patients with refractory focal epilepsy. For reducing the number of ICs to an optimal number by the minimum description length (MDL) criteria, the temporal dimension of the time series was reduced using principal component analysis (PCA). Then, the component activation maps were generated with *Z*-scoring the component voxel values and using the threshold of *Z* > 5. The results showed that the selected ICs, regardless of the spike presence during EEG recording, truly correspond to the epileptic activity. Considering only one of the ICs as the epileptic IC according to the overlap with the already known SOZ, the component activation maps were ordered. The average overlap between the epileptic IC and the SOZ was 10.6% ± 7.2.

Rummel et al. (103) analyzed the ordinal patterns and revealed that the BOLD responses to EEG-ICA predictors involved the brain region whose resection led to seizure freedom. In the study of Panda et al. (104), the EEG microstates were considered as the regressor in the GLM design to reveal the epileptic resting-state network. The EEG microstates were obtained from the maxima of the global field power (GFP) due to the stability in topography around the peaks of the GFP using sLORETA software. Considering each EEG microstate as an event, an input function was modeled based on the timing of each microstate and convolved with three columns customized gamma HRF. This model was added as the regressor in the GLM design for the ICA of fMRI data. The results of this method on five patients with epilepsy showed that using EEG microstate and ICA of fMRI data may examine the brain areas involved in resting-state brain discharge.

In another study (105), eight patients with epilepsy and known epileptogenic zone from the outcome of surgery were studied for the association between the ICs of fMRI epochs during the presence and absence of the IEDs. The fMRI data were divided into two epochs according to the EEG signal with visible IEDs and without IEDs. Then, spatial ICA was applied to each epoch separately, and IC maps were compared to the resection area and the EEG–fMRI correlation pattern by calculating a spatial correlation coefficient for identifying the epilepsy-related IC. The results showed a high similarity between the epilepsy-related ICs of the epochs with IEDs and those without IEDs. So, the epilepsy-related components are not contingent on the existence of the IEDs in the EEG signals.

Hunyadi et al. (106) studied 12 patients with refractory epilepsy and good surgical outcomes. The epilepsy-related independent components (eICs) were obtained from temporal ICA applied to EEG and spatial ICA applied to fMRI. After convolving the time courses of EEG ICs with the canonical HRF and upsampling the time courses of fMRI ICs to match the sampling rate of the EEG, Pearson's correlation coefficient was calculated for all possible pairs of EEG–fMRI ICs and labeled as matched for the correlation coefficient > 0.1. The results showed matching EEG-eIC for a single fMRI-eIC in four patients with three overlapped to the epileptic zone and matching EEG-eIC for at least two fMRI-eICs in six further patients.

Carnì et al. (107) compared two data-driven methods based on sICA and semi-blind ICA with the conventional GLM-based method using the EEG–fMRI data of 10 patients with epilepsy. A cross-correlation analysis was then completed between the epilepsy-related ICs and a GLM regressor. The results showed a concordance of the BOLD activation areas in response to synchronized epileptic activity obtained from sICA and semi-blind ICA with the GLM analysis and presumed electroclinical hypothesis. Semi-blind ICA showed more power against the noise and a higher correlation with the GLM regressor.

**Figure 6:**
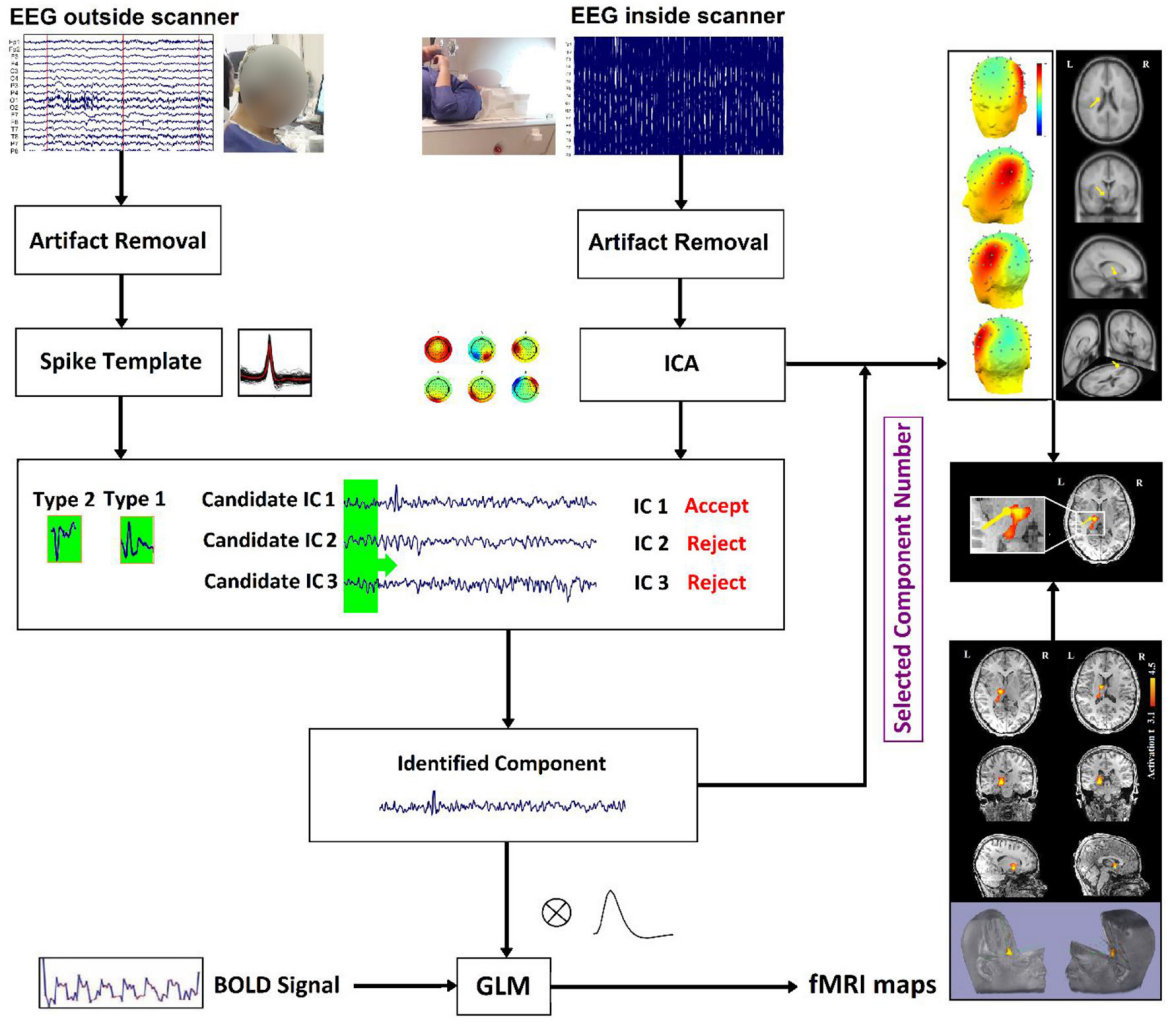
The model of the proposed approach in (22) to identify the components.

In our study (22), to measure the physical distance between BOLD clusters and selected component dipole location using patient-specific high-resolution anatomical images, we recommended a component-based EEG–fMRI method. The EEG–fMRI data of 17 patients with refractory focal epilepsy underwent this method for the localization of epileptic focus, determination of quantitative concordance, and comparison of the maximum BOLD cluster with the recognized component dipole. For the concordance level, the distance from the voxel with maximal z-score of maximum BOLD response to the center of the extracted component dipole was measured. This improved the localization accuracy to 97% that marks a significant rise compared to conventional works. Figure 6 illustrates a graphic illustration of the recommended technique in (22) to identify the components. The results of the implementation of the proposed method are shown in Table 1.

**Table 1:** Summary of IED studies which indicated a significant component-related BOLD response to consensus IEDs (22).

**Pt.-type**	**Ictal EEG**	**Interictal EEG**	**IED**	**Activation**	**Deactivation**
1-1	Temporal left	Temporal right/left	14	Temporal right/left (++)	–
1-2	Frontocentral bilateral	Frontal left	9	Frontocentral bilateral (*)	
2-1	Unclear	Frontal right	11	–	Frontal right (++)
3-1	Parietal left/right	Parietal left	13	–	Parietal right (+)
3-2	Temporal left	Parietotemporal left	8	Parietotemporal left (++)	Frontotemporal left-right (*)
4-1	Bilateral generalized	Bilateral generalized	9	Thalamus (++)	–
5-1	Unclear	Temporal right–left	15	Temporal right (*)	Temporofrontal (+)
5-2	Frontal right/left	Frontal right	6	–	–
5-3	Frontal left	Frontocentral left	7	Frontal left (++)	–
6-1	Left hemisphere	Frontotemporal left	17	Frontotemporal left (++)	–
7-1	Occipitotemporal right	Occipitotemporal right	12	–	Occipital right (++)
7-2	Bitemporal	Bilateral temporal	7	Bilateral temporal (++)	Bilateral temporal (++)
7-3	Left parietal/post temporal	Left parietal/post temporal	11	Paritotemporal bilateral (*)	Frontal right (–)
8-1	Frontopolar right	Frontocentral right	14	Frontal right–left (+)	Central right (–)
9-1	Unclear	Temporal right	12	Temporoparietal right (*)	Temporal right (++)
9-2	Parieto-occipital left	Parietal left	9	Parietal left (++)	Occipital right (–)

In the superscript, the topographical concordance between clinical localization and BOLD response is given: (++): same area, ipsilateral; (+): same area, contralateral; (*): ipsilateral but a different area; (–): no concordance (22).

**Figure 7:**
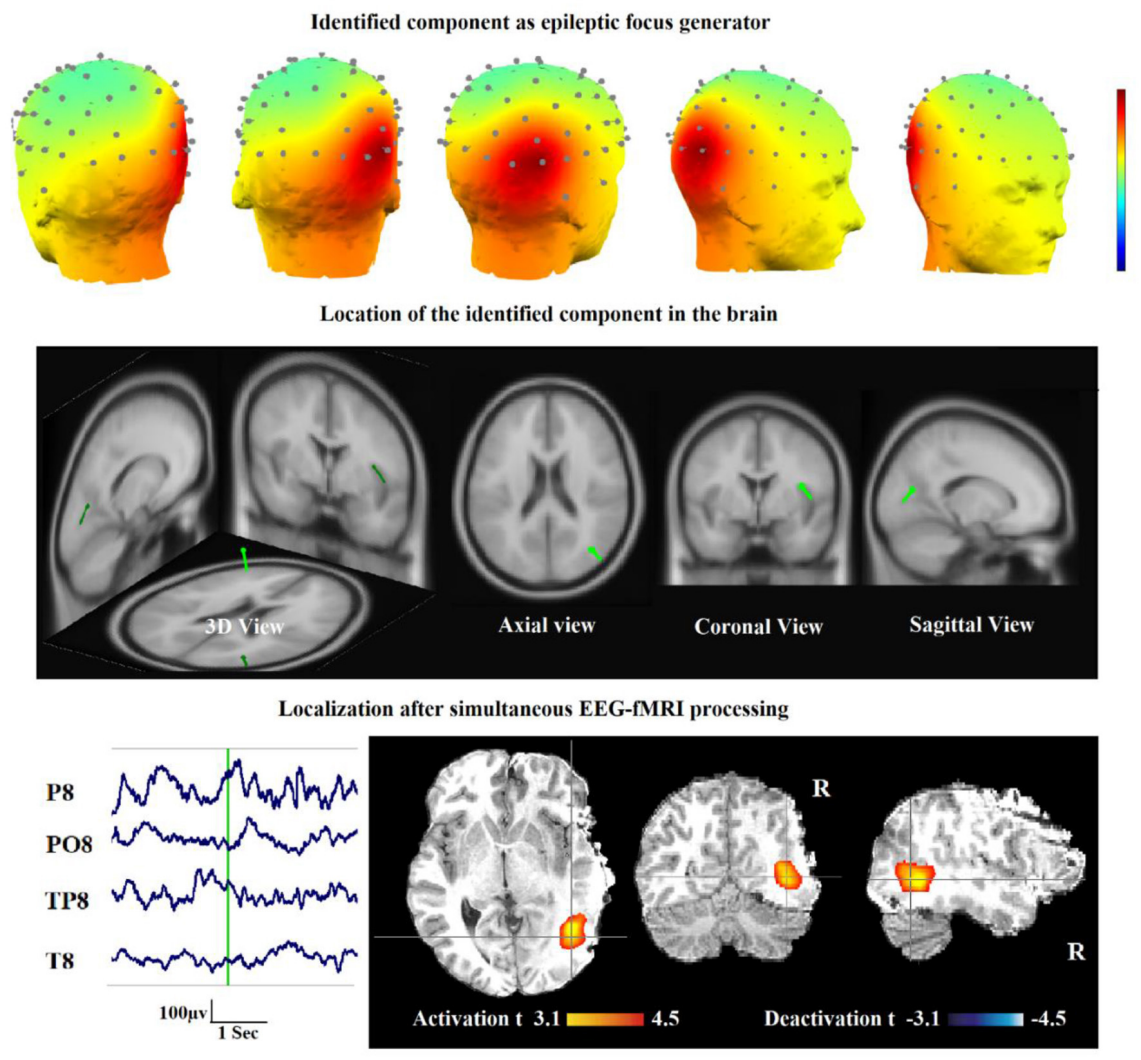
A sample of component-related BOLD response illustrates a neocortical activation in the first occipito-temporal cortex concordant with the spike field. Also, the marked events are in P8, PO8, and TP8 with referential montage. Top: The identified epilepsy-related component located in the right occipito-temporal lobe. Middle: The result of dipole-based localization of the identified component. Bottom: The localization of the epileptic generator acquired from simultaneous EEG–fMRI analysis (23).

Also, in our recent work (23), we found and obtained the time series of components associated with epileptic foci from EEG and added them to the GLM analysis. Twenty patients with refractory epilepsy and 20 age- and gender-matched healthy controls were studied, and the identified components were examined statistically to find the epilepsy-related components. The threshold of localization accuracy was determined as 86% using receiver operating characteristic (ROC) curve analysis, and the accuracy, sensitivity, and specificity were found to be 88, 85, and 95%, respectively. Also, the contribution of EEG–fMRI and concordance between the location of maximal BOLD response and the spike field were evaluated. The result confirmed the concordance in 19 patients and contribution in 17. Besides, considering the spatial correlation between the spike template and candidate components as well as the patients' medical records makes it possible to predict the behavior of epileptic generators. Figure 7 shows the results of the method proposed in (23), comparing three different methods. In this study, the epileptic focus localization can be viewed through the ICA algorithm, dipole, and on the MR images.

#### Dynamic Causal Modeling Analysis

Dynamic causal modeling (DCM) is another useful tool that can be used for estimating the synaptic drivers of cortical dynamics during an epileptic seizure. However, it has a costly computation in the requisite Bayesian inversion procedure (108).

In the study of Hamandi et al. (109), the EEG–fMRI data was acquired from a 23-year-old patient with refractory TLE. The EEG spikes were detected and convolved with an HRF and its temporal derivative to be used as the onsets of GLM. The results showed activation related to the left anterior temporal interictal discharges, in the left temporal, parietal, and occipital lobes. For determining the functional relationship between the IED-related activation areas, DCM was used and the deployment of neural activity from the focus of temporal to the region of occipital activation was suggested. Also, for tractography analysis, the probabilistic index of connectivity (PICo) algorithm was used to detect the anatomical connections of TL activation and showed connections from this origin to the site of occipital activation, which delineate the pathways of deployment of epileptic activity.

In a study of Murta et al. (110), the EEG–fMRI data from five patients with focal epilepsy were analyzed for detecting the focus of epileptic seizures. For this purpose, three different methods were used, and the outcomes were compared with the clinical outlook: (1) the classic method based on GLM at different neurophysiology regressor lags (LasgM) considering 19 regressors by lags ranging from −16 to +20 s in 2-s steps around the events which were convolved with four types of HRF including single gamma with its temporal derivative, canonical HRF with its temporal derivative, gamma bases functions, and FIR basis functions. In this method, the activation map was obtained using GLM analysis for each lag, and the lags with the maximum number of activated voxels for each VOI were selected to detect the focus of activity propagation; (2) the DCM method, which is a suitable model-based method for studying effective connectivity and has been used several times in the fMRI data of epilepsy patients (109, 111); and (3) the Granger causality (GC) which is a data-driven statistical hypothesis test to analyze effective connectivity in fMRI data with the primary precondition of stationary covariance for the data variables (62, 112–114). Evaluating the results of three methods revealed that DCM analysis, although suffering from generally poor SNR, provides meaningful results in a sufficient number of seizure events. Also, the LagsM results were concordant with the clinical anticipation as much as to be a useful complementary approach. However, the CG results showed that this method seems to be not appropriated to use in the cases like this effective connectivity analysis, at least with the situation of SNR and time resolution of the data used in this study.

**Figure 8:**
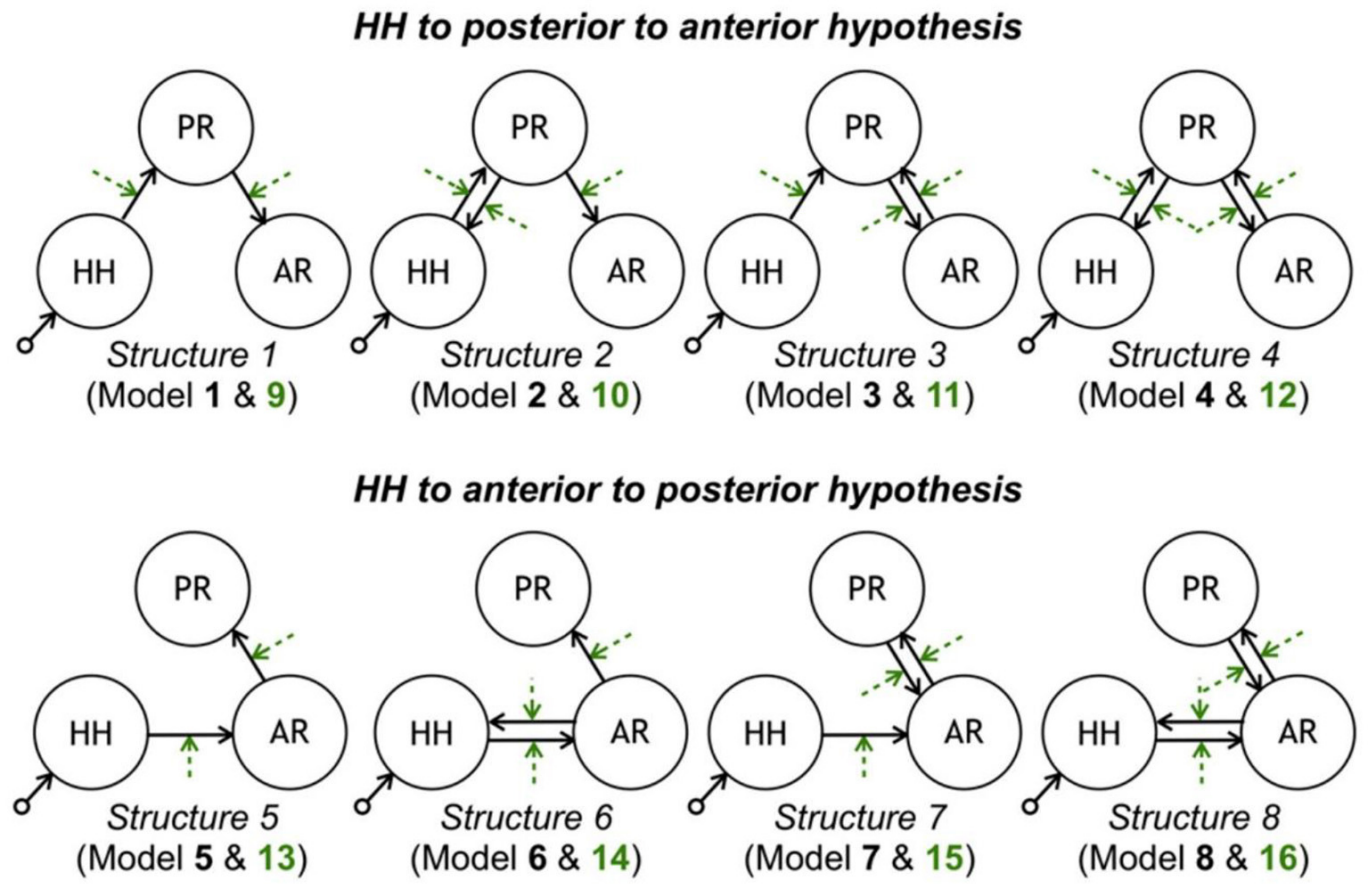
Model space tested with DCM. Each row contains eight models consistent with each propagation hypothesis. Each column corresponds to a different latent connectivity structure. For each latent connectivity structure, the linear model is presented with solid arrows and the bilinear model is presented with solid arrows (intrinsic connections) and dashed arrows (connections' modulation). Seizure activity is fed into the HH network node (115).

In epilepsy associated with hypothalamic hamartomas (HH), although the origin of seizures is known to be in HH, diffusion pathways are not known specifically. Murta et al. in (115) employed the DCM approach to estimate these diffusion pathways from the fMRI data acquired from an HH patient. Examination evaluating a set of clinically possible network connectivity models of discharge diffusion, the most likely model to explain the data showed a diffusion pathway from the HH to the temporal–occipital lobe followed by the frontal lobe. Therefore, this method makes it possible to find the diffusion pathway of seizures, which is helpful in the surgical procedure of epilepsy treatment (Figure 8).

Vaudano et al. in (116) studied a patient with reading epilepsy (RE) to identify the network of seizures. The BOLD, significant changes were obtained in 21 s around each seizure corresponding to various linear combinations of a set of Fourier basis functions to find a range of possible HRF shapes. Then, using the results of this analysis, four ROIs were selected, and four linear models were constructed using DCM to analyze the effective connectivity between ROIs. It was eventually revealed that the dominant premotor cortex (BA6) is the origin of seizures in RE, but also an area in the left deep PFC is closely linked to the beginning of the epileptic activity.

For the localization of epileptic focus, IED-related fMRI maps acquired from common analysis methods often show a network including multiple regions of the signal change instead of a highly focal region that drives the generation of seizures within the epileptic network. Vaudano et al. in (117) used the DCM approach to identify the SOZ on the EEG–fMRI data of one patient with FLE. Although pre-surgical EEG–fMRI showed two distinct clusters of IED-related BOLD activation in the left frontal pole and the ipsilateral dorsolateral frontal cortex, the DCM approach revealed the left dorsolateral frontal cortex as the driver of changes in the frontopolar area, and An et al. in (118) generated the BOLD activation maps and linearly registered them to postoperative anatomic MRI images for 35 patients with focal epilepsy who later had a surgical resection. The results showed 10 fully concordant patients with maximum *t*-value inside the resection area, nine partially concordant patients with maximum *t*-value near to resection area and overlapped results, five partially discordant patients with a less significant cluster inside the resection area, and 11 fully discordant patients with no response related to the resection area.

#### Functional Connectivity Analysis

Functional connectivity is a perfect technique for epilepsy to detect the complex brain effects because of dysfunctional and maladaptive networks produced by seizures (119).

Preti et al. in (120) recommended a new way to reveal the connectivity changes associated with an epileptic activity using the information of EEG and dynamic functional connectivity (dFC). Applying this method to the EEG–fMRI data of two patients with epilepsy revealed the specific patterns of connections and disconnections successfully associated with the epileptic activity.

Omidvarnia et al. (121) studied seven patients with focal epilepsy who underwent EEG–fMRI to identify the relationship between the interictal EEG power and local fMRI connectivity. The wavelet coherence was developed between dynamic regional phase synchrony (DRePS, calculated from fMRI) and band amplitude fluctuation (BAF) of a target EEG electrode with dominant IEDs. This approach revealed the regions with a concordance between EEG power and local fMRI connectivity that were near the suspected SOZ in some of the cases. Also, the found regions had a little overlap with the results of conventional EEG–fMRI analysis more in medial posterior cortices, perhaps because of reflecting different aspects of the epileptic network.

**Figure 9:**
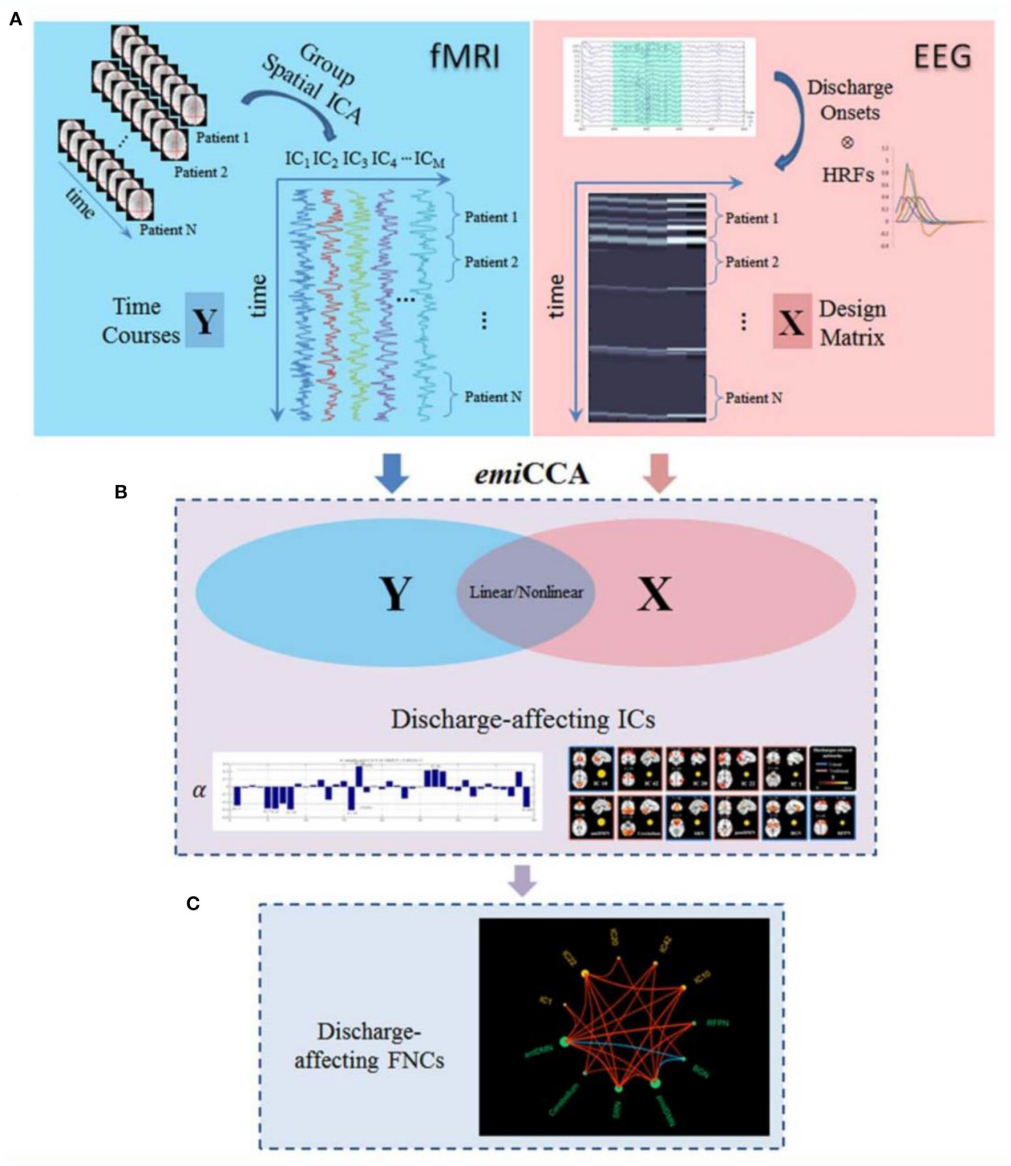
The framework of discharge-affecting network analysis using emiCCA. **(A)** Dataset Y was defined by applying group ICA to fMRI data and concatenating the ICs across the patients. Also, after identifying the onsets of GSWDs by neurologists and convolving with four SPM canonical HRFs peaking at 3–9 s, one Glover HRF, and one single Gamma HRF, a design matrix containing all of them formed the dataset X. **(B)** The emiCCA was applied for identifying significant linear and non-linear discharge affecting ICs with weights (α) exceeding the 1.5 standard deviations of weight values corresponding to the significant maximal information Eigen coefficients (MIECs). **(C)** For examining the possible functional network connectivity between the networks identified by emiCCA, the maximal time-lagged correlation method was used (122).

In a study of Dong et al. (122), 18 patients with juvenile myoclonic epilepsy (JME) were studied to identify discharge-affecting networks using eigenspace maximal information canonical correlation analysis (emiCCA) and functional network connectivity (FNC) analysis (Figure 9). emiCCA is a data-driven method to detect the linear and non-linear relationships between two datasets, which can be the EEG discharges and fMRI networks in JME, and tackle the multivariate problem in the comparison of two datasets (123). Also, the FNC is an approach to identify the interactions between resting-state networks (RSNs) and the effects of epileptic discharges on them (124–127). The results showed a relationship of the epileptic discharges with the discharge-affecting networks in the DMN, self-reference (SRN), basal ganglia (BGN), and frontal networks. Also, a significant increase was found in FNCs between the salience network (SN) and resting-state networks.

In the study of Siniatchkin et al. (128), the EEG–fMRI data recorded from 33 children with focal and multifocal epilepsy during sleep and resting-state functional connectivity were acquired using 15 ROIs. For the focal epilepsy patients, some strong correlations were found between the corresponding interhemispheric homotopic regions with a short-distance and weak long-distance functional connectivity similar to the healthy children. However, for the multifocal epilepsy patients, significantly stronger correlations were found among several regions of DMN, thalamus, and brainstem with longer-distance functional connectivity and not dependent on the presence of Lennox-Gastaut syndrome in patients.

In another study (129), a total of 261 IED events from 21 patients with unilateral left and right TLE were identified, and a 20-s period around them was used in the dynamic FC analysis for left and right hippocampus and amygdala separately. The results showed that the left IEDs had more effect on the hippocampus-seeded networks and caused FC changes in the reward–emotion network (more of the prefrontal-limbic system) and visual network, but the right IEDs had more effect on amygdala-seeded networks and caused a coactivation in the reward-emotion network (more of the reward system).

**Figure 10:**
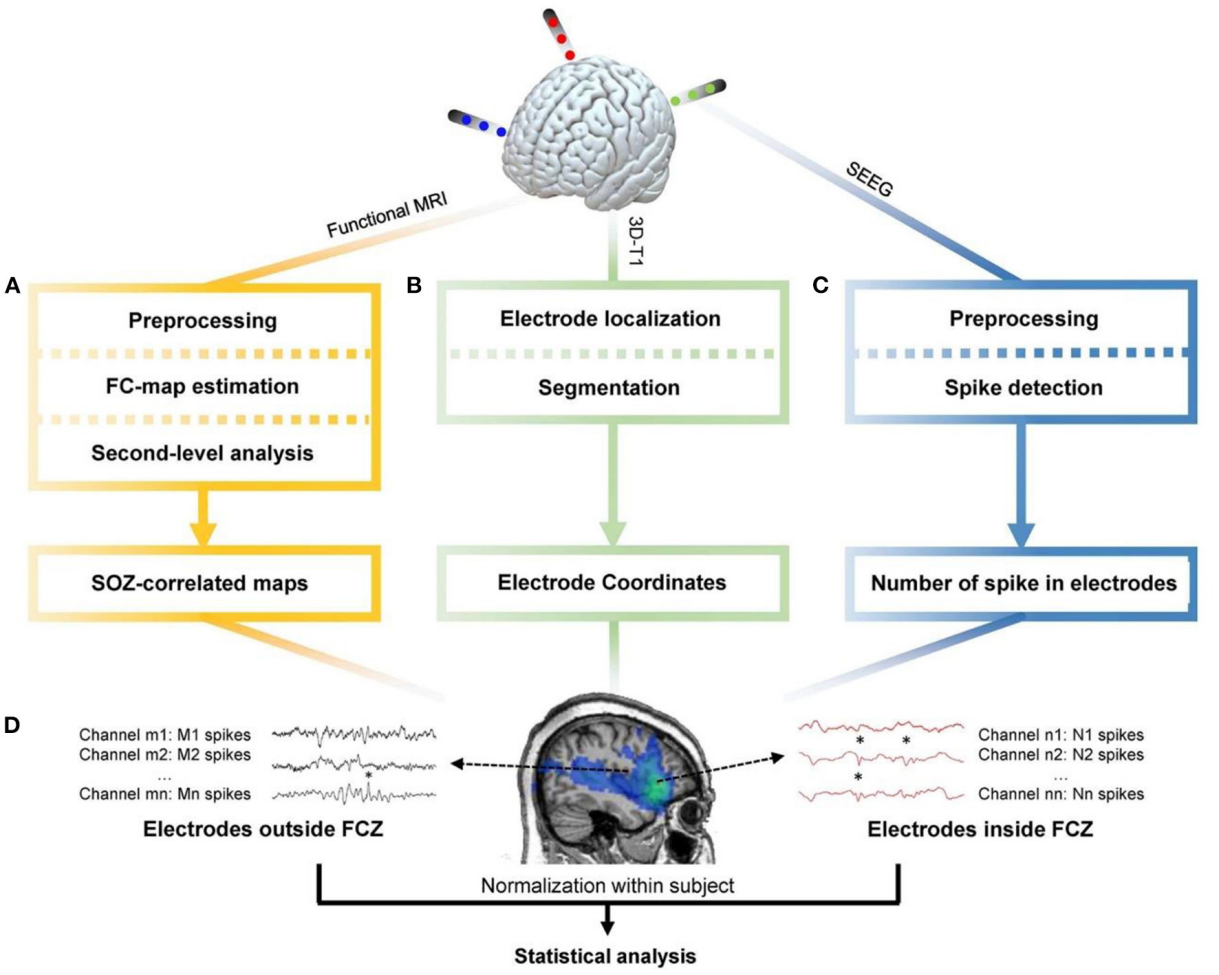
The general pipeline of the research (130). **(A)** Functional MRI data were preprocessed through realignment, slice timing, outlier detection, coregistration, segmentation, spatial smoothing, and noise regression. Then the maximal BOLD response and its neighboring 26 voxels were used as seed regions to calculate the seed-based functional connectivity maps. One-sample *t*-test was applied to determine regions with significant functional connectivity. **(B)** Preimplantation 3D-T1 images were segmented to obtain the brain region. The postimplantation 3D-T1 images were first coregistered to the pre-implantation images, and then the location of electrodes was determined. **(C)** The iEEG data were resampled to 200 Hz, band-pass-filtered between 10 and 60 Hz, and notch-filtered at 60 Hz to eliminate noise. Spike detection based on signal envelope distribution modeling was applied afterward. **(D)** The number of IEDs in each channel was normalized by the median number of IEDs in each subject. Statistical analysis was performed to determine group difference of IED rates between channels inside and outside the FCZ (130).

Su et al. (130) identified the different types of IEDs according to the spatial distributions from 38 patients with focal epilepsy and were used separately in the analysis of IED-related BOLD responses. The concordance between the maximal BOLD responses and the SOZ was found using iEEG, and then the functionally connected zone was determined for each one using the maximal BOLD as a seed (Figure 10). Lastly, IED rates in iEEG channels inside and outside the functional connectivity zone (FCZ) were examined. The results of 36 studies from 25 patients revealed that IED rates inside the FCZ were considerably greater than outside in concordant cases.

**Figure 11:**
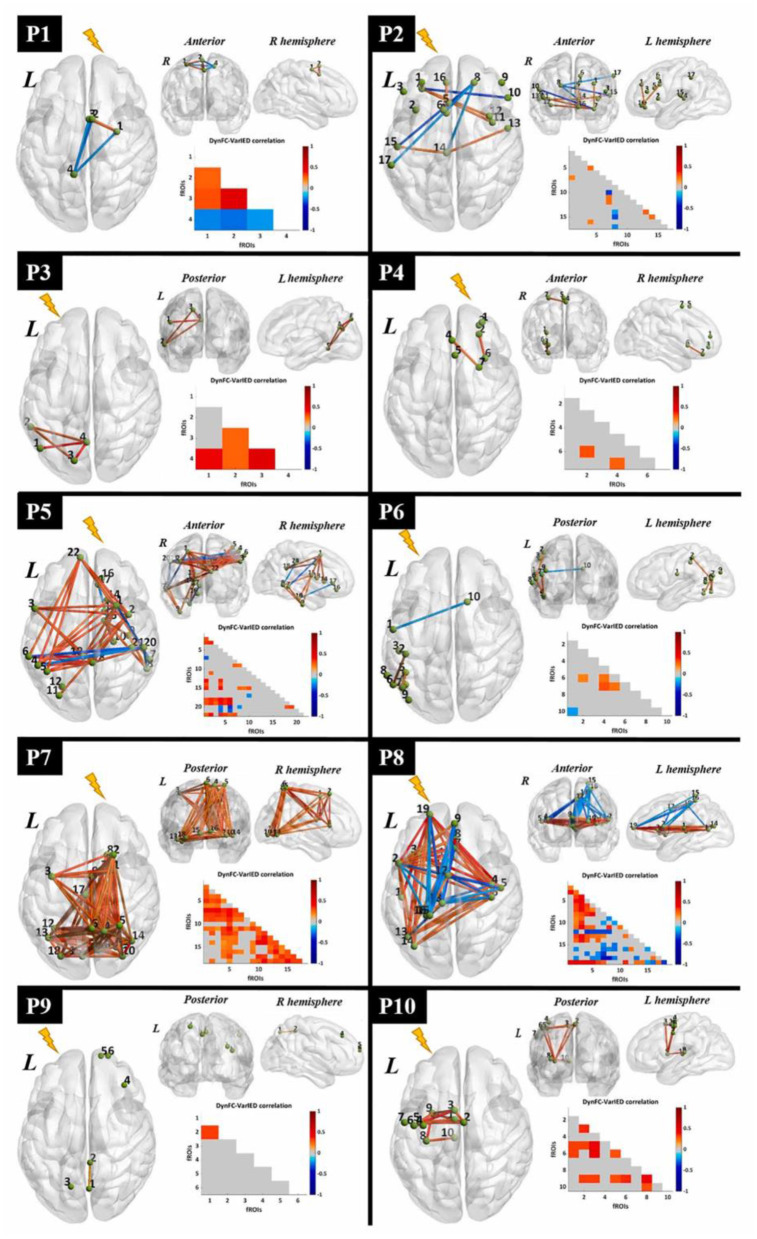
Visualization of dynamic epileptic subnetwork. For each patient, the dynamic epileptic subnetwork is shown in the form of a brain graph in axial, coronal, and sagittal views. Green spheres of equal size represent fROIs, labeled with a number indicating their statistical relevance in the epileptic network. The strength of significant connections is color-coded according to a global color bar scaled in the range [−1, 1]. The dynamic epileptic subnetwork is also reported in the form of a lower triangular correlation matrix with equivalent color-code. The lightning bolt indicates the epileptogenic hemisphere for each patient. L, left; R, right (131).

In a study of Iannotti et al. (131), 10 patients with pharmacoresistant focal epilepsy were studied, and the regions involved in epileptic network generation were identified by GLM analysis using the time course of fMRI-defined focus acquired from the IED-related BOLD maps as the main regressor. Then, using a sliding-window approach, the dFC time courses were assessed between the involved regions and correlated with the sliding-window variance of the IED signal (VarIED) to identify connections whose dynamics related to the epileptic activity. This method's results revealed the epileptic network in nine patients with dynamic subnetwork connections proximate to the epileptic focus (Figure 11).

#### Electrical Source Imaging

Electrical source imaging (ESI) is a non-invasive, low-cost method of localizing the sources of the EEG signals recorded with scalp electrodes (132). So, it also can be used in the EEG–fMRI analysis of localizing the epileptic sources.

In the study of Vulliemoz et al. (133), 13 IED types detected from nine patients with focal epilepsy were used as the separate regressors in the GLM to obtain the map of IED-related BOLD signal changes. Also, in 12 cases, the electrical source imaging (ESI) could be performed successfully on the IEDs using a realistic head model (SMAC) and a distributed linear inverse solution (LAURA). The results showed that in 10/12 studies, ESI at IED onset (ESIo) was anatomically close to one BOLD cluster in which, for 4/12, it was most relative to the maximally significant positive BOLD cluster, and for 4/12, it was closest to the negative BOLD responses. Furthermore, in 6/12, ESI at a later time frame (ESIp) revealed a diffusion to remote sources co-localized with other BOLD clusters. So, this study showed that analyzing ESI and EEG–fMRI simultaneously can discriminate areas of BOLD response related to the initiation of IED from propagation areas.

In another similar study of Vulliemoz et al. (134), the maps of BOLD responses explained by continuous activity of the estimated IED sources (cESI) were compared to the results of the conventional IED-related analysis. The comparison showed a concordance between the results in 13/15 different types of IED. The cESI model showed other major BOLD alterations in the concordant regions for 10/15, better detection of the IED-related BOLD responses in 4/7, and contaminated diffusion pattern due to the incompletely corrected artifacts of the source signal in four IED types.

Brodbeck et al. (135) performed the ESI using LAURA on the IEDs of 10 operated patients with non-lesional MRI, and at postsurgical follow-up of at least 1 year five had extratemporal lobe epilepsy. The results showed localization of the SOZ in eight patients correctly, and it means that ESI reflects the definite source of the epileptic activity. However, the spike peak comprises the diffusion areas.

In another study (136), nine children with refractory focal epilepsy undergoing pre-surgical evaluation were studied. The resected area was compared with three analyses for the localization of epileptic foci, which were, respectively, the conventional method, the analysis of IED-related BOLD changes using spike-specific voltage maps of average IED acquired from long-standing monitoring outside the scanner, and the ESI approach using LAURA. The concordant results of activation within the resection area using the mentioned analysis were revealed in three, four, and all the nine patients, respectively. Therefore, the ESI method is a more valid approach to localize the epileptic foci in children with refractory focal epilepsy.

**Figure 12:**
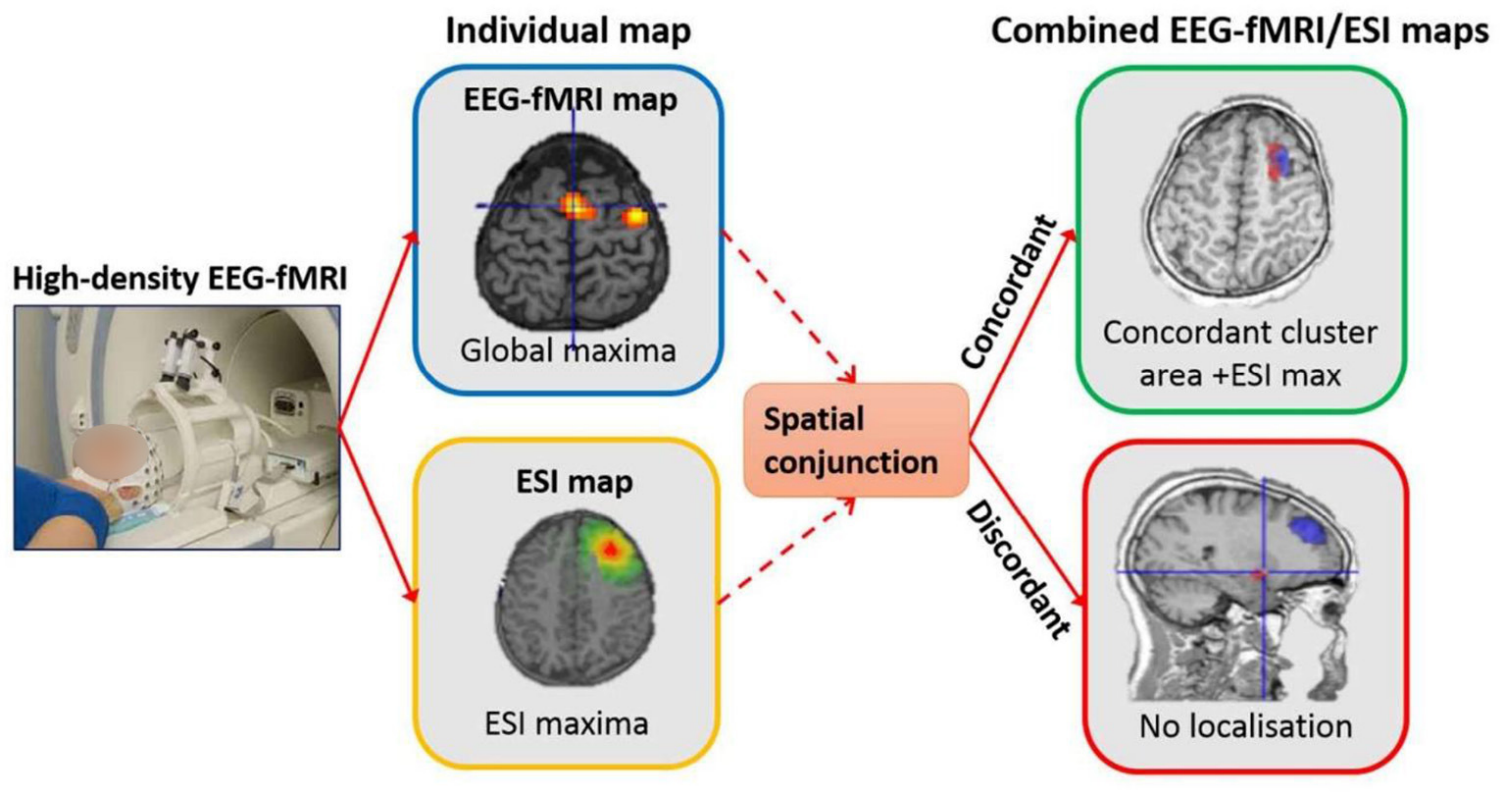
Localization extraction procedure from left to right. For the individual map of EEG–fMRI, the result of localization based on global maxima is shown. Also, for the individual map of ESI, the same result based on maxima in the map from the 50% rising phase of the IED is shown. For the combined test, the spatial conjunction of both maps was used for the localization. Only for the concordant maps, the result of combined localization was extracted from the region encompassing the ESI max and the closest significant EEG–fMRI cluster located in the same sub-lobe (137).

Also, Centeno et al. (137), studied 53 children with drug-resistant epilepsy, and the localization map of the epileptic focus was performed using BOLD responses, ESI, and the combination of both maps. Comparing the results with the presumed epileptic focus and the postsurgical outcome revealed significant maps in 52 patients, which included 47 for EEG–fMRI, 44 for ESI, and 34 for both. Also, the epileptogenic zone was concordant with the results of 29 patients, which included 11 for EEG–fMRI, 17 for ESI, and 11 for both (Figure 12).

### Long-Term EEG Recording

In conventional methods, an experienced neurophysiologist reviews the EEG obtained from within the scanner and identified and marked the timing of epileptiform discharges. Spikes were modeled as zero-duration events, convolved with a standard HRF, and used as a regressor for the GLM model and fMRI analysis (138). Given that it is difficult to detect spikes inside the scanner due to artifacts, many studies have suggested automatic detection methods. These methods require long-term EEG recording outside the scanner (139, 140). In many studies, to extract the spike pattern inside the scanner, it is necessary to identify the spike pattern of the same subject outside the scanner in order to extract the spikes inside the scanner through computational methods and detection algorithms (22, 24, 83). To this end, IED-related spikes distinguished on the EEG collected outside the MRI scanner are averaged to build a patient-specific spike template, and their similarity is then examined through methods such as cross-correlation (23, 141, 142). In these studies, all patients undergo a preoperative assessment at the hospital, including long-term monitoring (143). To evaluate the extracted results from source localization algorithms, the results obtained need to be compared with the medical results obtained from different modalities. For the localization of SOZ and irritative zone (IZ) in the pre-surgical evaluation of each patient, all the available data such as the comprehensive clinical record, full neurological examination, long-term video-EEG monitoring (144), structural MRI (145), neuropsychological assessment, and other non-invasive investigations such as PET and ictal SPECT (146) are usually reviewed.

An important study by Grouiller et al. (139) benefited from long-term EEG recording to localize seizure foci in patients without inside scanner IEDs. To this end, the correlation of epilepsy-specific EEG voltage maps with the hemodynamic changes was investigated in 23 patients with focal epilepsy. An epilepsy-specific EEG voltage map was built by averaging IEDs acquired from long-term clinical EEG recording outside the scanner. Then, for each time frame, the correlation between the voltage maps of the EEG signals outside and inside the scanner was calculated. Next, the time course of the correlation coefficient convolved with a standard HRF was used as a regressor for fMRI analysis. The results of this technique were like those of the conventional analysis in all five patients who had significant BOLD changes associated with IEDs. More importantly, the method correlated BOLD responses with the scalp maps of epileptic activity in 14 out of the remaining 18 patients who had inconclusive simultaneous EEG–fMRI study using conventional analysis due to the absence of IEDs in the inside scanner EEG recording.

In another study (147), 30 patients with drug-resistant TLE and undergoing TL resection were monitored. The IEDs were visually identified by experts on the intra-MRI EEG, and the average topography map of IEDs recorded during long-term video-EEG outside the scanner was computed. Then, both of them were used as the regressors of a GLM analysis, and the results of BOLD responses in TL were divided into two groups of Concordant and Discordant compared to the surgical resection areas. So, it was revealed that 13 of the patients with good surgical outcomes were in the concordant group (16 patients), and only three of them were in the Discordant group (14 patients).

**Figure 13:**
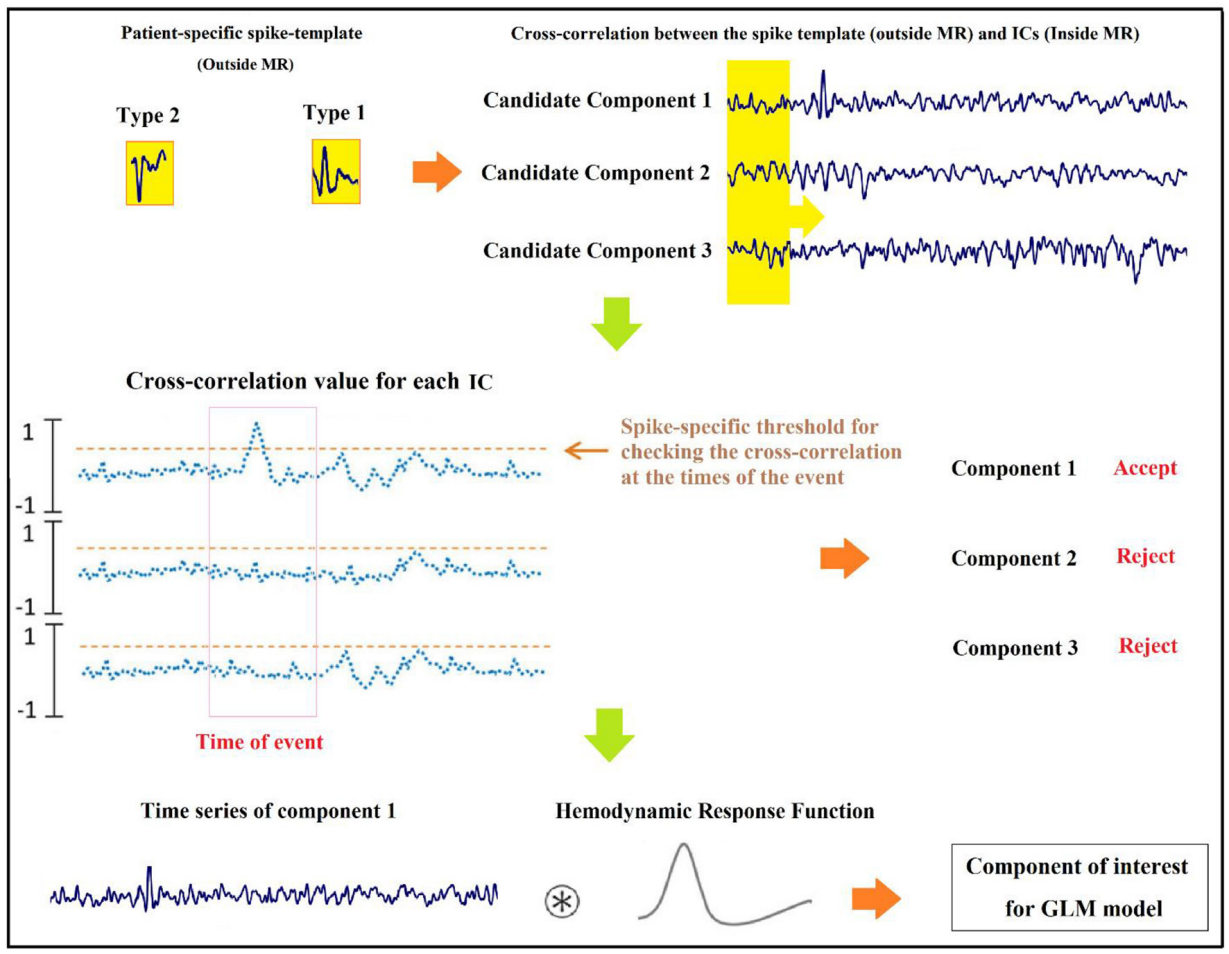
Graphic illustration of the suggested method for identification of components (24).

In our previous study (24), we extracted the IED template from the outside of the scanner for computing the correlation. To this end, IED-related spikes were detected in the outside of the scanner and were averaged to build a patient-specific spike template. After band-pass filtering, the template was ultimately outlined by a significant spike deflection on the EEG channels, beginning from the onset at baseline to the negative peak of the following slow wave. The objective was to identify the neural behavior of epileptic generators by detecting the components-of-interest and using the GLM analysis substituting in the classical linear regressor. The general pipeline of this study is shown in Figure 13. This method applied 28 IED sets from nine patients who were excluded for surgery because of the unclear focus in four, presumed multifocality in three, and a combination of the two conditions in two of them. The results revealed at least one BOLD response, which was significant, positive, and topographically related to the IEDs in eight patients.

### Localization of Epileptic Focus Using Other Approaches

#### EEG Slow-Wave Discharges

In the study of Laufs et al. (148), a patient with refractory epilepsy was studied using continuous EEG–fMRI, characterizing the seizures by head turning to the left and clonic jerking of the left arm that suggests a right mesial frontal onset zone. The routine interictal EEG showed symmetrical post-central alpha rhythm and occasional runs of independent, non-lateralized slow activity in the delta band with right frontocentral dominance. Although long-term scalp EEG, structural MRI, and the EEG during simultaneous EEG–fMRI showed no clear significance, the observed slow activity suggests a role for seizure localization with EEG–fMRI even in the absence of clear interictal discharges.

Manganotti et al. in (149) compared the BOLD signal changes on fMRI in two states of rest and activation in terms of EEG focal interictal slow-wave discharges. In all the eight volunteered patients with partial epileptic seizures, the EEG activation of focal slow-wave discharges caused a significant BOLD activation in the related brain region. This significant concordance showed that focal BOLD activation provides useful information for the pre-surgical process even in partial epilepsy patients whose standard EEGs demonstrate focal interictal slow-wave discharges without spikes.

#### Additional EEG Measures

In the recent EEG–fMRI studies for identifying epileptic focus, some patients have shown poor sensitivity and inconsistency between EEG epileptic foci and BOLD activation patterns. That said, using additional measures may be helpful for better localization of epileptic focus. Moehring et al. (150) studied 11 children with focal epilepsy. Then, the sleep-specific activities such as sleep spindles, k-complexes, and vertex sharp waves were extracted, characterized as a twig function, convolved with a canonical HRF peaking at 6 s, and considered in the GLM as the additional separate regressors. The results showed that considering these regressors increased the significance of activated voxels inside the anticipated IED source area and decreased the number of significantly activated voxels outside of it. So, using the sleep-specific activities in the statistical model is useful to achieving better sensitivity and results of identifying seizure foci in epilepsy.

**Figure 14:**
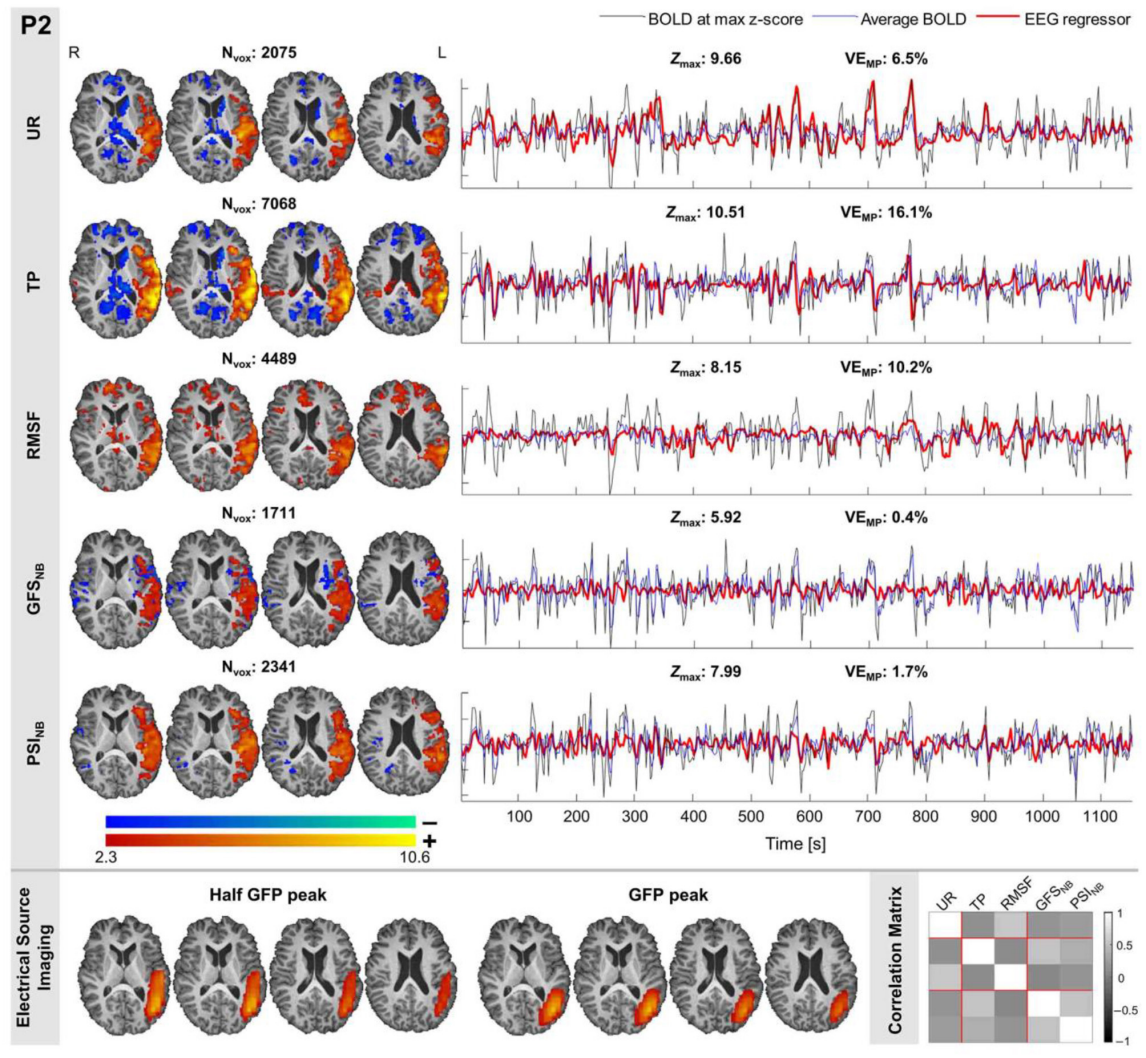
The results of epileptic network mapping for a patient. (Top–Left) The epileptic networks obtained using the EEG regressors UR, TP, RMSF, GSF_NB_, and PSI_NB_, together with the number of voxels (Nvox); the color codes red-yellow and blue-green depict positive and negative BOLD responses, respectively. (Top–Right) The BOLD signal measured at the maximum Z-score voxel (black trace), the average BOLD signal within the activation cluster (blue trace), and EEG regressor (thicker red trace), together with the maximum Z-score (Zmax) and the variance explained by the motion parameters (VEMP). (Bottom–Left) ESI solution maps at IED onset and propagation, obtained at half the maximum of the first rising phase of GFP and its associated peak, respectively, for validation of the GLM-derived epileptic networks. Consistent results with the ESI solutions were obtained for all patients with clear IEDs only when using the PSI_NB_ metric. (Bottom–Right) Correlation matrix between all metrics of interest (151).

Also, in the study of R. Abreu et al. (151), the phase synchronization index (PSI) and global field synchronization (GFS) within the frequency bands of 1–45 and 3–10 Hz along with the root mean square frequency (RMSF), total power (TP), and conventional unitary regressors were computed and used to reveal the associated epileptic networks on nine EEG–fMRI datasets including IEDs. After cross-validating the results through ESI, the best performance was revealed using the average PSI within 3–10 Hz across several measures in all datasets (Figure 14). Also, testing the PSI in three patients with no IEDs during EEG recording showed partially reasonable networks in all patients.

#### Mutual Information Maps

The most outstanding feature of using mutual information (MI) for the EEG–fMRI analysis is the balance of involving both imaging modalities, not requiring any prior model of HRF or relationship between EEG spikes and BOLD responses (152).

In the study of Caballero Gaudes et al. (153), five patients with epilepsy underwent EEG–fMRI and electroclinical localization of epileptic focus. For each IED onset, a period with TR duration was defined, and the result was downsampled to the temporal resolution of BOLD signals. Then, the voxel-wise MI was computed between the EEG–fMRI score and the fMRI data, and MI maps were thresholded using a non-parametric wavelet resampling approach. Comparison of the results with the electroclinical localization and conventional GLM-based analysis revealed a concordance of focal BOLD responses in four patients.

**Figure 15:**
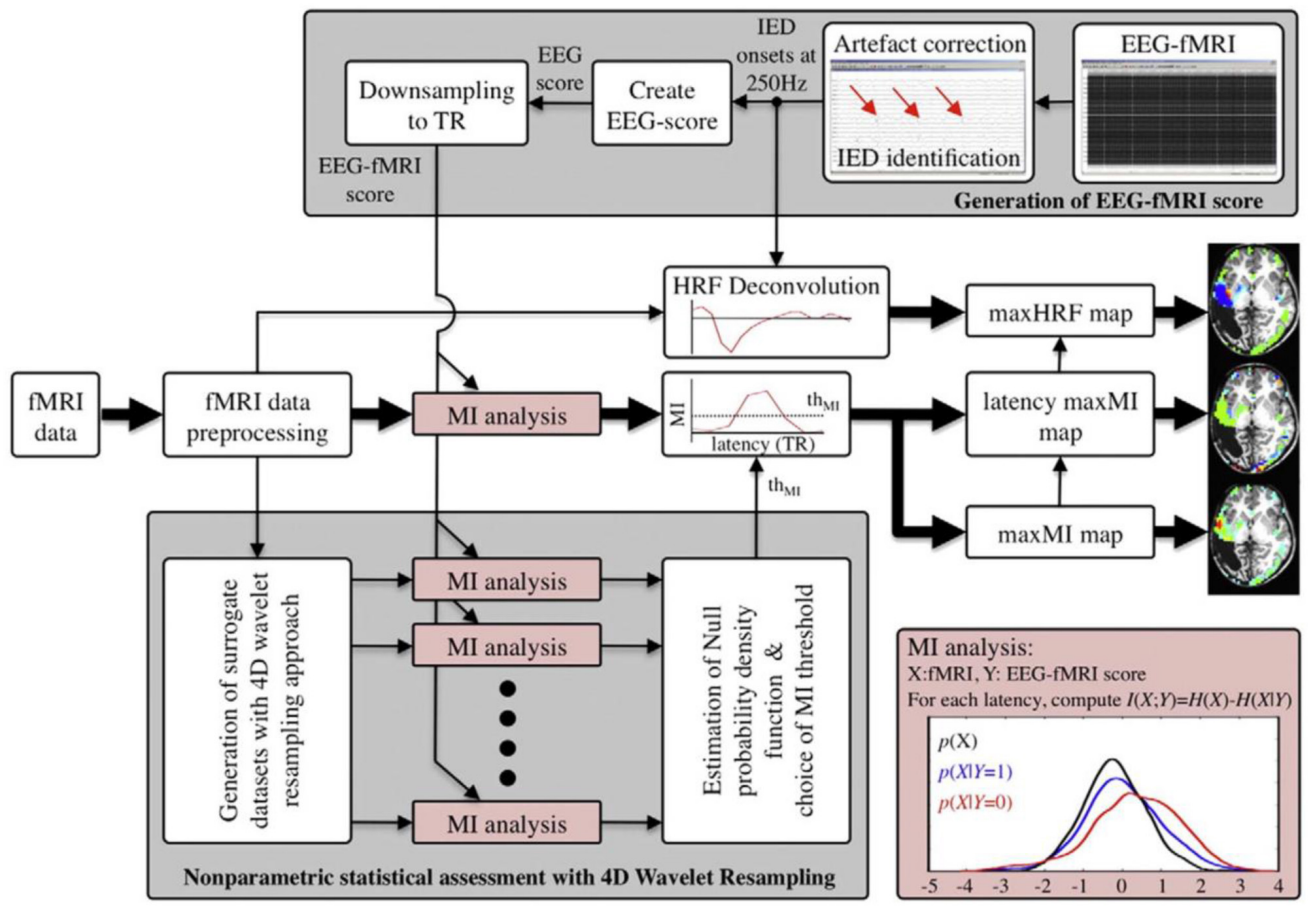
Schematic diagram of the information-theoretic approach. The EEG recorded in the MR scanner is corrected for gradient and pulse artifacts. The time of occurrence of IED peaks is marked, and the EEG score indicating the existence of epileptic activity is created and finally downsampled to the temporal resolution of fMRI (TR) to generate the EEG–fMRI score (top gray-shaded square). The fMRI data is first preprocessed (rigid-body registration for motion correction, spatially smoothed, high-pass filtered, and z-normalized). The MI between the fMRI voxel time series and the EEG–fMRI score is computed based on the entropy and conditional entropy (bottom-right red-shaded square) at multiple latencies by shifting the EEG–fMRI score, resulting in an MI time course. The shape of the HRF is deconvolved based on the IED timing. Significant MI statistics are those exceeding a thresholded th_MI_, which is chosen according to the non-parametric statistical procedure where 19 surrogate datasets created with a 4D wavelet resampling approach are analyzed in the same way as the original dataset and the PDF of the MI statistics under the null is estimated (bottom gray-shaded square). To summarize the results, three maps are generated: a maximum MI map, a latency map showing the latency at which the maximum MI occurs, and a map plotting the amplitude of the HRF at the latency of the maximum MI (154).

Caballero-Gaudes et al. (154) investigated the MI between the IEDs on EEG and BOLD signal on fMRI to generate the MI maps and validate its performance for the localization of epileptic focus (Figure 15). The EEG–fMRI data of 14 patients with pharmacoresistant focal epilepsy were used to generate the MI maps based on the four-dimensional wavelet packet resampling method. Comparing the results with the statistical maps obtained from two conventional GLM methods showed the same concordance of ~57% with the epileptogenic area defined electro-clinically or surgically.

#### Voxel-Based Morphometry

In the study of Salek-Haddadi et al. (155), nine patients with reading epilepsy underwent simultaneous EEG–fMRI with an extra recording of voice, electromyography (EMG), and electrocardiography (ECG), and six of them experienced reading-induced seizures during recording. Also, 30 neurologically normal control subjects with a similar age range and gender distribution were scanned for comparison. Voxel-based morphometry (VBM) was used for the structural brain analysis. However, as the result of VBM analysis, no significant differences in gray matter density were detected comparing the epilepsy patients with the control group.

#### Non-linear Hemodynamic Responses

Pouliot et al. (34) studied the EEG–fMRI data recorded from three patients with refractory focal epilepsy for quantifying non-linear hemodynamic responses using the second-order expansion of the Volterra kernel. In the Volterra expansion, which is a functional Taylor expansion, the time-dependent inputs were epileptic spikes, and the outputs were BOLD, oxyhemoglobin (HbO), and deoxyhemoglobin (HbR) time series at a certain fMRI voxel. The results showed significant non-linearities in all the patients with a good concordance to the epileptic focus and negative BOLD response regions. Furthermore, this method identified the epileptic focus in one patient who had shown nothing while common analyses.

#### Two-Dimensional Temporal Clustering Analysis

The two-dimensional temporal clustering analysis (2dTCA) is a data-driven approach for the localization of epileptic networks using fMRI data. Maziero et al. in (156) used the EEG–fMRI data of 14 patients with epilepsy as inputs to the 2dTCA for generating the histograms and adding to GLM as predictors. The results showed success in eight patients, not confined to the presence of IEDs, while the conventional analysis identified coherent maps in only six patients who had at least one IED during recording.

Maziero et al. (157) also used the 2dTCA to map the seizure onset zone in 18 patients with focal epilepsy (12 presenting IEDs). The results of this method, along with the conventional method, were compared to the region of surgical resection. The concordant results showed that 2dTCA was successful in localizing the EZ in 13 patients (3 of the cases with no IEDs), but the conventional method was successful in only five of the patients who presented IEDs.

#### Lateralization Index

Mangalore et al. in (158) used the EEG–fMRI data of 10 patients with refractory epilepsy who showed well-formed IEDs in a proposing method to lateralize the seizure focus in an ROI with the aid of the peak BOLD signals. For each patient, the lateralization index was computed from the significant clusters of different ROIs using the following formula: the number of activated voxels multiplied by the Z-scored intensity of activation in the given ROI. Then, the seizure focus was determined by thresholding the lateralization index. Compared with the output of other modalities, the results of this method were successful in temporal and extratemporal lobe epilepsy, reflex epilepsy, and lesional epilepsy. The only disadvantage of EEG–fMRI in this work was if irrelevant BOLD changes were correlated with the specified IED or not.

#### Adapted Directed Transfer Function

**Figure 16:**
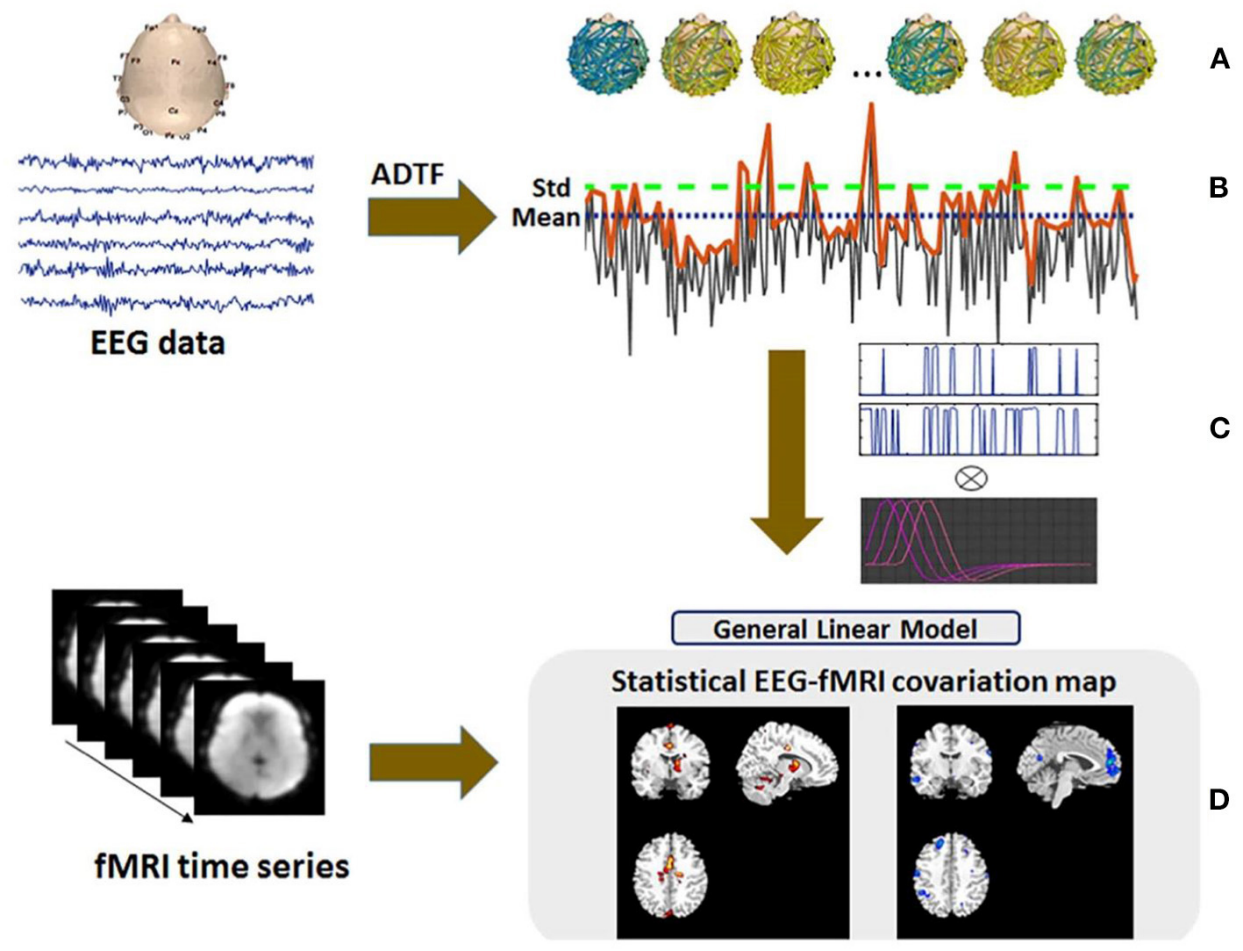
An overview of the suggested EEG–fMRI analysis. **(A)** After preprocessing the EEG signal, the time-varying scalp network was constructed using ADTF. **(B)** The variation of the ADTF information flow between electrodes in each 2-s time window was extracted for the generation of network variation time series. **(C)** The significant values of network variation time series exceeding one standard deviation and the mean were selected as the regressors and added to the GLM analysis, respectively. **(D)** The results were acquired from the GLM analysis (159).

In the study of Qin et al. (159), 18 patients with juvenile myoclonic epilepsy (JME) underwent simultaneous EEG–fMRI. Between EEG electrodes, the adapted directed transfer function (ADTF) values were computed to describe the time-varying network, and its information within sliding windows were used as a temporal regressor in GLM analysis (Figure 16). The outcomes demonstrated that BOLD activations allied with high network variation were mostly placed in the thalamus, cerebellum, precuneus, inferior TL, and sensorimotor-related areas, including the middle cingulate cortex (MCC), supplemental motor area (SMA), and paracentral lobule. Also, the deactivations related to medium network alternative were originated in the frontal, parietal, and occipital areas.

#### Four-Stage Localization Method

Wan et al. (160) proposed a four-stage method for the localization of SOZ that includes identifying events of interest using Hilbert transform, acquiring channels of interest (CoIs) using the Shannon-entropy-based complex Morlet wavelet transform (SE-CMWT)-based power spectral density, detecting high-frequency oscillations (HFOs) on CoIs with the combination of adaptive-genetic-algorithm-based matching pursuit (AGA-MP) and Morlet wavelets, and localizing SOZs based on the half-maximum method using characteristics of HFOs. This approach showed the highest sensitivity and specificity compared to the four existing methods of SE-CMWT, AGA-MP, RMS, and CMWT.

### Ancillary Issues

#### The Relation Between rCBF and Epileptogenic Areas

Studies have shown that seizures induced by musical stimulation, especially in temporal epilepsy, cause a rise of regional cerebral blood flow (rCBF) in putative epileptogenic foci and the other brain regions. However, this is a virtual temporal relation between epileptic discharges and rCBF changes due to the offline EEG recordings (161). In the study of Marrosu et al. (161), simultaneous EEG–fMRI recording of musicogenic elicited seizures was studied in a patient with partial epilepsy. The statistical maps obtained from the GLM technique showed that EEG features extracted from epileptogenic areas are largely coupled with rCBF increase. Also, the rCBF changes in other areas may suggest further aspects of musicogenic seizures. For instance, this physiological activation induced by music in several brain areas may initiate musicogenic seizures in predisposed subjects.

#### Validation of EEG–fMRI Results Using a Gold Standard

**Figure 17:**
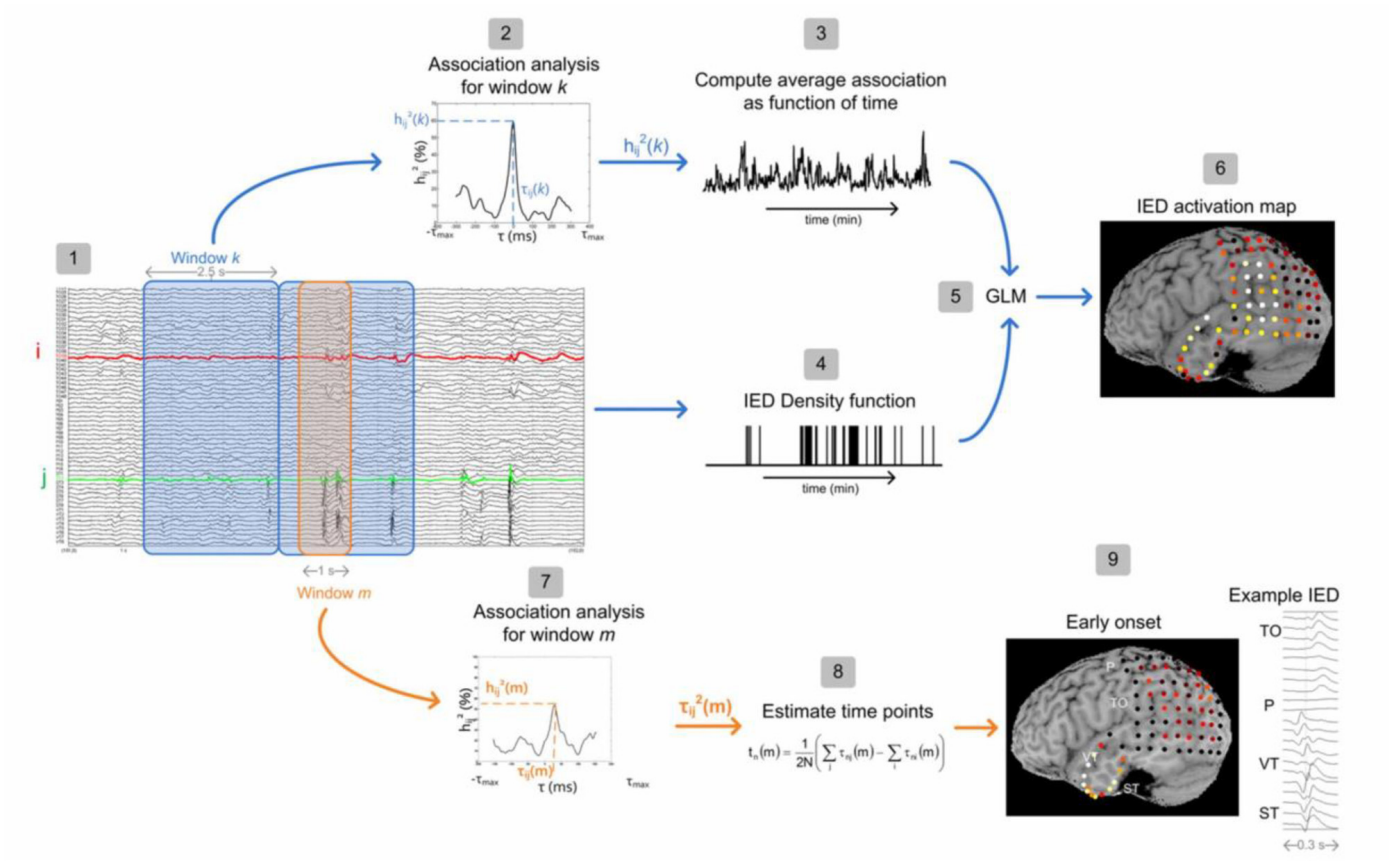
Flowchart of ECoG analysis consisting of two steps: estimation of interictally active ECoG areas (steps 1–6) and the estimation of an onset area (steps 7–9) (162).

For the validation of EEG–fMRI outcomes with a gold standard to figure out the actual role of this multimodal approach in pre-surgical evaluation, Houdt et al. (162) compared the correlation patterns of EEG–fMRI data acquired from 16 surgical candidates with the involved brain areas of ECoG IEDs, the SOZ, resected area, and degree of seizure freedom (Figure 17). The results of the comparison revealed a concordance between at least one of the EEG–fMRI areas and an interictally active ECoG area for all patients. Also, the EEG–fMRI areas covered the whole SOZ in 83% and resected area in 93% of the dataset.

#### The Relations Between IEDs and SOZ

Regarding the relations between IEDs and SOZ, Yamazoe et al. (163) hypothesized that the number of IEDs and their spatial extent could contribute to revealing the SOZ. To test this hypothesis, 157 types of IED grouped by spatial distribution were extracted clinically from the EEG–fMRI data of 64 patients with refractory localization-related epilepsy. Then, each IED was convolved with four HRFs peaking at 3, 5, 7, and 9 s to construct four regressors, and a combined *t*-map was created with the most significant *t*-value at each voxel. Two levels of significance were defined to observe reliable activation in the combined t-maps. The first level was defined by any set of five contiguous voxels with the *t*-value ≥ 3.1, and the second level was the *t*-values being higher than the whole-brain topological false discovery rate (FDR) of 0.05 for multiple-cluster comparisons. For each type of IED, the primary cluster was referred to as the cluster with the highest absolute *t*-value at a peak located in the cerebral cortex compared to the thresholds defined in significance levels. Finally, the presumed seizure onset zone (pSOZ) of the patients that were determined using SEEG findings or the other comprehensive evaluations (164) was compared to the primary cluster in EEG–fMRI to measure their concordance at the sublobar level. The result of this study confirmed the initial hypothesis and revealed the significance in the number of IEDs in the types with *t*-value above FDR that was higher than below FDR and in the extent of IED types concordant with the SOZ that was larger than IED types discordant with the SOZ. The complex pathophysiology of epileptic cerebral structures, types of seizures, and frequency features have not been studied as the authoritative factor for precise detection of epileptic foci using EEG–fMRI (22).

## Conclusions

Recording EEG and fMRI simultaneously is a non-invasive method identifying cerebral hemodynamic changes related to IEDs on scalp EEG. Several studies revealed the capacity of EEG–fMRI to distinguish various forms of generalized and focal epilepsy. In patients with epilepsy, especially those who are pharmacoresistant and surgical candidates, the significant clinical matter of how BOLD changes relate to IEDs can contribute to localizing the epileptic focus. The BOLD signal usually rises in regions causing focal IEDs, but often in the context of more extensive, or even distant, responses.

The simultaneous EEG–fMRI recording is an effective non-invasive method to study the brain regions associated with the epileptic discharges. The neuronal discharges that occur through interictal spikes or spike-wave bursts cause an increase in metabolism and blood flow, redirected in the BOLD signal measured by fMRI. Although this increase has the highest intensity in generating discharges, it can be revealed in areas only affected by the discharges. Also, the epileptic discharges can lead to a decrease in metabolism that the origin of which is not completely understood. It has been shown that EEG–fMRI applied to patients with focal epilepsy results in maxima of the BOLD signal most often concordant with other localization methods and helped to localize the epileptic focus in non-lesional frontal-lobe epilepsy. It has also been revealed that the thalamus is an active region in generalized epileptic discharges. These can be used to investigate the location and extent of the brain regions intricate during epileptic discharges and evaluate the disease progression.

Simultaneous recording of EEG and fMRI provides a great potential to find the pathophysiological mechanisms of the discharges (165). The most capable method of acquiring data is probably continuous scanning followed by EEG artifact removal. Some cases have shown inconsistent fMRI results with EEG. However, we cannot imagine a one-to-one correspondence between EEG and fMRI findings. These inconsistencies may be due to the fMRI data analysis problems. Some of the responses shown in the fMRI results are “noises” caused by practical artifacts such as movement, an erroneous HRF model, or inappropriate statistical methods. Despite the noise, most responses can be considered valid since they make sense in the context of our understanding of an epileptic condition.

It is also essential to consider the natural differences between the two modalities. First, in fMRI, the BOLD response is measured everywhere, but EEG records only superficial cortical layer activity. Secondly, two different types of activities are evaluated: one is electrical, and the other is based on the changes in deoxyhemoglobin in the veins. EEG and fMRI are considered complementary since each measures an activity that the other one does not.

Although the ideal approach of data analysis remains undefined, the majority of focal and generalized epilepsy patients had a consistent BOLD effect with the spikes. Instead of using techniques developed for functional activation in the future, there should be a focus on adapting fMRI analysis techniques to the specific requirements of the epileptic activity. Friston et al. (166) proposed a method that does not depend on linear assumption. Other approaches such as temporal clustering try to analyze the BOLD signal independently of the EEG event (167, 168). The deconvolution approach makes assumptions with regard to HRF (169, 170). Finally, the ICA approach decomposes the data sets into spatially independent components. Using some of these methods, we may be able to discover epileptic discharges anywhere in the brain, regardless of seeing spikes on the scalp EEG.

The importance of the diverse BOLD response is another issue that should be assessed. In epilepsy studies, the fact that we see both activation and deactivation is considered perplexing, where it is expected to see activation (increased BOLD) as a result of extreme neural activity. Moreover, it is important to assess particular responses in different types of epileptogenic structural abnormalities such as mesial temporal sclerosis, brain tumors, and malformations of cortical development (MCDs), which are commonly complicated by intractable focal epilepsy (171).

The presence of both positive and negative BOLD responses in generalized epilepsy patients may be interpreted differently, also indicating the explanation of deactivation. Bilateral activations were observed in the thalamus, mesial mid-frontal region, insulae, and cerebellum. Deactivations were found bilaterally in the anterior frontal and parietal regions, in a global pattern resembling the default state of the brain (98). This finding suggests that the default state of the brain is suspended during an epileptic discharge. Deactivation occurs as a result of the indirect effect of the discharges on attention mechanisms. Performing these studies on experimental animals provides further insight into human results (172, 173).

When the BOLD responses are found in multiple regions, particularly in focal epilepsy, this possibility arises that the regions are related to the propagation of the interictal discharge, or distant sites particularly sensitive to the effect of epileptic discharges. However, the temporal resolution of fMRI is not able to measure the propagation times of a few milliseconds. So, the EEG source modeling can help to assess the propagation of epileptic discharges if the model includes EEG sources in the same regions as BOLD responses. BOLD response patterns may be different in the primary epileptogenic region and in the region in which the activity propagated (42). It would be interesting to assess functional connectivity using the fMRI data (174).

In the past, most studies used a 1.5-T scanner, although a few studies used 3 T. Using a 3-T scanner may create the expectation of better recognition of hemodynamic changes and deteriorating some difficulties such as higher signal loss as a result of susceptibility artifact, the pulse artifact, and movements that cause worse artifacts in the EEG. Fortunately, with suitable artifact removal methods, studies in a 3-T scanner would be more efficient (26).

Finally, an important study of Markoula et al. (175) assessed the impact of EEG–fMRI on the clinical decision-making process and showed the actual capability of this approach to be applied prospectively in localization of seizure focus during the pre-surgical evaluation. They studied 16 patients with refractory extra-temporal focal epilepsy, referred for pre-surgical evaluation in a period of 18 months. Interpretable EEG–fMRI results which were available in 13 patients made a modification of the initial surgical plan in 10 (77%), suggesting a significant influence of EEG–fMRI on epilepsy surgery planning.

In conclusion, combining EEG and fMRI seems to be a potential method in the source localization of epileptic foci. This complicated technique is quite practical and offers a new view in the study of epileptic disorders. Although applying it to individual patients (subjects) to localize epileptic foci is not yet justified, it can present potential areas for further research, for instance, focused anatomical MRI analysis or electrode implantation.

All in all, the works reviewed in this paper can bring us closer to the localization of focal epileptic activity and, afterward, to real-life applications. Applying simultaneous EEG–fMRI for combining EEG temporal resolution and fMRI spatial resolution recommends more excellent diagnoses of precise epileptic source localization. This allows for providing more patients with the option of surgery while increasing the likelihood of a successful and life-improving operation.

## Author Contributions

SS, EE, and HS-Z jointly designed the study. SS, EE, and MSh did the literature survey and wrote the initial version of the manuscript. HS-Z edited the draft and submitted the manuscript. All authors participated in the revision process and approved the final version of the manuscript.

## Conflict of Interest

The authors declare that the research was conducted in the absence of any commercial or financial relationships that could be construed as a potential conflict of interest.
